# The oxidative stress regulator, PerR, is required for staphyloferrin B-mediated iron acquisition in *Staphylococcus aureus*

**DOI:** 10.1128/jb.00149-26

**Published:** 2026-07-14

**Authors:** Alexander A. Sheikh, Ronald S. Flannagan, Nathan J. Nicholson, Natalie Jarycki, Racheal O. Fabelurin, Ian A. Lewis, David E. Heinrichs

**Affiliations:** 1 Department of Microbiology and Immunology, University of Western Ontario 6221, London, Ontario, Canada; 2 Alberta Centre for Advanced Diagnostics, Department of Biological Sciences, University of Calgary 2129, Calgary, Canada; University of Illinois Chicago, Chicago, Illinois, USA

**Keywords:** iron homeostasis, siderophores, iron regulation, MRSA, *Staphylococcus*

## Abstract

*Staphylococcus aureus* is a globally prevalent gram-positive pathogen that can cause numerous types of infection. Due to host nutritional immunity and iron (Fe) sequestration, *S. aureus* experiences Fe limitation during infection. To overcome this, *S. aureus* expresses an arsenal of Fe acquisition systems whose expression is coordinated through the Fe-binding transcriptional regulator, Fur. Here, from a screen to identify *S. aureus* mutants defective for Fe-restricted growth, we identified several with mutations in *perR*, encoding a transcriptional regulator involved in resistance to oxidative stress. RNA-seq identified that the most downregulated genes in a *perR* mutant growing in Fe-restriction are those from the *sbn* operon that encodes staphyloferrin B biosynthesis. In agreement, *perR* mutants grew poorly in Fe-deficient media due to deficient staphyloferrin B production. In a subcutaneous model of *S. aureus* skin infection, *S. aureus perR* caused significantly smaller lesions, consistent with our finding that this mutant had decreased alpha-hemolysin expression during Fe-restricted growth. These findings are consistent with the hypothesis that PerR acts to fine-tune access to Fe ostensibly to avoid Fe-dependent toxicity. The importance of the PerR function to *S. aureus* was further highlighted by examination of over 8,000 human bloodstream isolates of *S. aureus*, showing that the PerR sequence was highly conserved. Together, these findings demonstrate the importance of PerR to *S. aureus* in providing an additional level of regulation of Fe homeostasis beyond Fur-dependent Fe sensing.

**IMPORTANCE:**

*Staphylococcus aureus* is a notoriously antibiotic-resistant human pathogen and has the potential to cause a myriad of potentially life-threatening infections. Understanding the intricacies of *S. aureus* pathogenesis in the host will underpin the development of novel therapeutic approaches that target bacterial virulence mechanisms rather than essential cellular processes. The significance of our research lies in identifying how *S. aureus* regulates Fe acquisition in response to environmental signals, providing further insight into nutrient sensing and acquisition at the host–pathogen interface to facilitate the development of therapeutics that may target this process.

## INTRODUCTION

The gram-positive bacterium *Staphylococcus aureus* is both a human commensal and a leading cause of nosocomial infection (1). *S. aureus* infections can manifest as relatively minor skin and soft tissue infections or as serious life-threatening illnesses, such as bacteremia or staphylococcal toxic shock syndrome (2, 3). The ability of *S. aureus* to cause varied clinical conditions can be attributed, in part, to its capacity to colonize diverse tissues and adapt to ever-changing environments within the host; it does so by sensing diverse cues and eliciting changes in gene expression. Indeed, the nutritional status of the bacterial cell is an important cue, and it has been established that the regulation of nutrient acquisition, metabolic, and virulence genes is intimately linked (4–6).

During infection, nutrients, including trace metals such as iron (Fe), are actively sequestered by the host through a process termed nutritional immunity (7). Fe is a critical nutrient required for growth by virtually all bacteria, and Fe acquisition is of critical importance to *S. aureus,* as evidenced by the fact that the bacterium encodes approximately 60 proteins dedicated to a role in acquiring Fe in any one of several different chelated forms (8). These include the *cnt* locus that codes for the biosynthesis, efflux, and transport of a Fe^2+^-binding metallophore, staphylopine (SP), or the *isd* locus coding for a high-affinity heme uptake and utilization system (9, 10). However, the majority of Fe acquisition genes present in the *S. aureus* genome encode proteins involved in the production, efflux, and uptake of siderophores, which are secreted low-molecular-weight high-affinity Fe^3+^-binding molecules (11). *S. aureus* encodes four characterized transport systems for the uptake of siderophores (12). The endogenously synthesized polycarboxylate siderophores staphyloferrin A (SA) and staphyloferrin B (SB) are taken up by protein products of the *hts* and *sir* loci, respectively (13, 14). The *sfa* locus (for SA) and the *sbn* operon (for SB) code for the biosynthesis and efflux of these siderophores by *S. aureus* (13, 15). In addition, *S. aureus* can utilize hydroxamate and catechol(amine)-type siderophores that derive from other sources due to the activity of the Fhu and Sst transporters, respectively (14, 16). Combined, these systems allow *S. aureus* to utilize a broad repertoire of siderophores that may exist within varied niches. For instance, *S. aureus* might favor SB over SA-mediated Fe acquisition in glucose-replete environments because carbon catabolite repression impedes citrate production that is required for SA synthesis (17).

While Fe acquisition is vital to the growth and survival of *S. aureus*, excessive Fe uptake through unregulated import can be toxic (18). Consequently, the expression of Fe acquisition and metabolism genes in *S. aureus* is subject to complex transcriptional regulation. For instance, the repressor protein Fur (Ferric uptake regulator) binds to a conserved operator sequence, called a Fur box, within promoters of Fe-regulated genes to prevent transcription during growth in Fe-rich environments (19, 20). Additional regulatory factors allow for fine-tuning of Fe-regulated gene expression; these include, but are not limited to, the SbnI protein that positively regulates transcription of the *sbn* operon in a heme-dependent manner, and the non-coding regulatory RNA *isrR,* which controls the expression of several genes that function in Fe metabolism, including heme synthesis (21, 22). Furthermore, expression of genes involved in Fe homeostasis is also modulated in response to intracellular reactive oxygen species (ROS) through the repressor protein PerR. PerR, like Fur, binds to a conserved operator sequence known as a PerR box within the promoter–operator of genes related to ROS detoxification (e.g., *katA*, *ahpCF*) and Fe storage (e.g., *ftn, dps*) to prevent transcription when ROS are scarce. However, under conditions of ROS abundance, PerR undergoes histidine oxidation, thereby altering its ability to remain bound to DNA (23–26). Oxidation of PerR is catalyzed by a bound Fe(II) cofactor, linking ROS sensing with Fe in the cell.

Here, we report an as-yet unrecognized role for PerR in modulating the ability of *S. aureus* to grow under conditions of Fe restriction. Using an unbiased forward genetic screen in a *S. aureus* strain lacking characterized Fe acquisition systems, we leveraged the function of the Fe and oxygen-dependent antibiotic streptonigrin (SN) to select for SN^r^ mutants presumably further defective in Fe acquisition. Through this process, we identified several SN^r^ *S. aureus* variants with mutations in *perR*. Further characterization revealed that, under Fe-restricted growth conditions, *S. aureus perR* mutants were unable to grow, a phenotype we showed was attributed to perturbed SA- and SB-mediated Fe acquisition. In keeping with the role of PerR as a transcriptional regulator, we show that *perR* mutants exhibit major transcriptomic changes under conditions of Fe restriction, including decreased expression of toxins and proteases but most notably significant downregulation of the SB biosynthetic operon (*sbn*). *In vivo*, PerR was shown to influence *S. aureus* pathogenesis in a murine subcutaneous infection model, and, in a cohort of over 8,000 *S. aureus* human bloodstream isolates, the *perR* gene was highly conserved relative to other global transcriptional regulators.

## RESULTS

### *S. aureus perR* mutants have heightened resistance to SN

**Fig 1:**
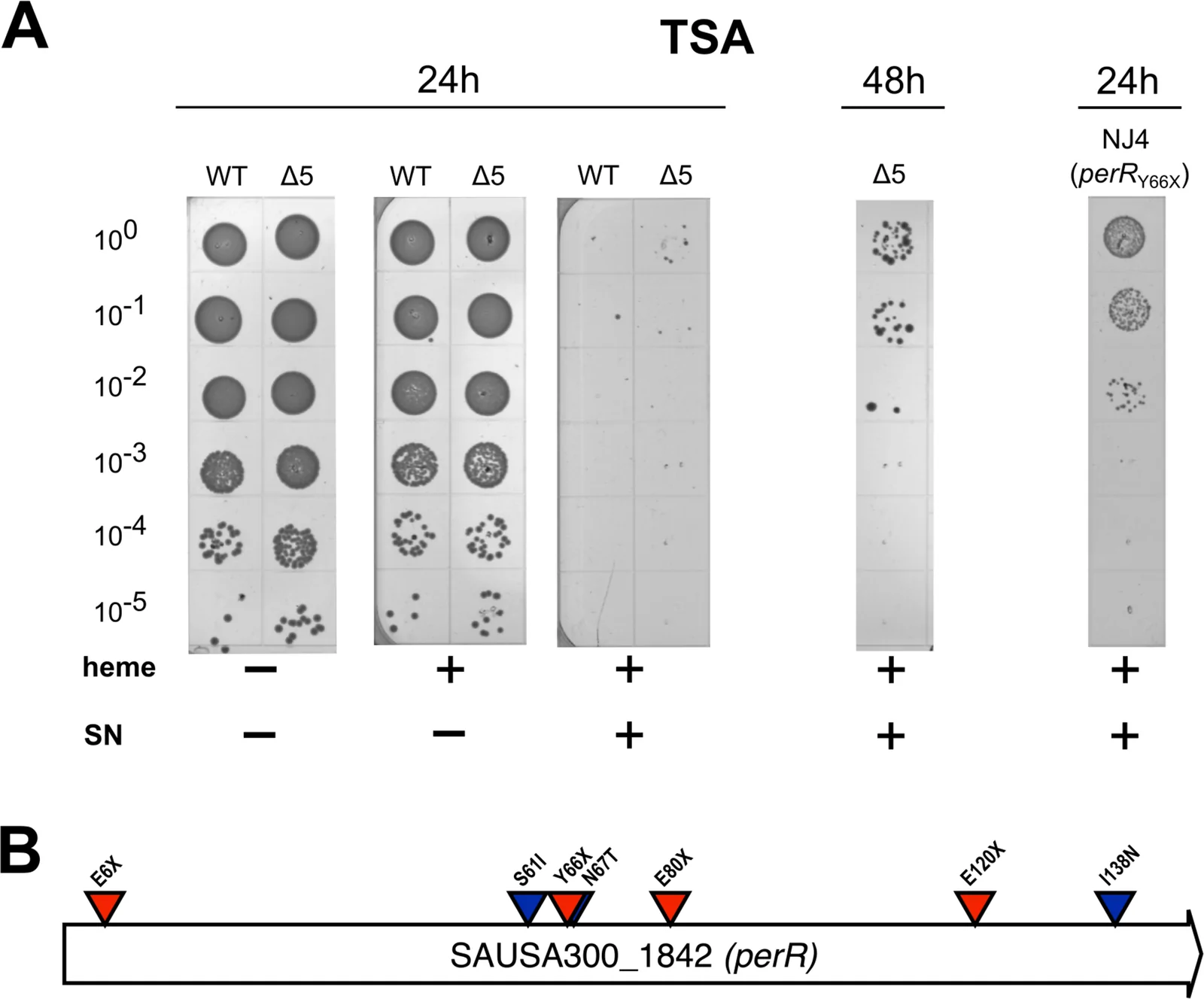
Mutation of *perR* provides increased resistance to streptonigrin. (**A**) Growth of WT, Δ5 parent strain, and a SN-resistant derivative of Δ5 (NJ4, containing a Y66X SNP in *perR*) on TSA containing one or both of 1 µM bovine hemin and 200 ng/mL SN. (**B**) Location of the various *perR* SNPs obtained through screening and their corresponding amino acid changes. Red labels represent a nonsense mutation, and blue labels represent a missense mutation. Labels correspond to one or more resistant isolates.

Hypothesizing that there were as-yet undefined mechanisms of Fe acquisition in *S. aureus*, we generated a mutant, called Δ5, that is defective for five of the characterized Fe acquisition systems (SA biosynthesis [*sfaABCD*], SB biosynthesis [*sbnABCDEFGHI*], SP biosynthesis [*cntKLM*], ferric hydroxamate uptake [*fhuG*], ferric catechol(amine) uptake [*sstABCD*]) and used this mutant to select mutations affecting Fe-dependent growth. SN, whose antibacterial activity is dependent on intracellular Fe, was used to select for mutants with enhanced SN resistance, as Fe transport mutants can display increased SN resistance (27–29). Thus, the use of Δ5 instead of WT USA300 ensured that we would not re-identify the major characterized Fe acquisition systems. When plated on tryptic soy agar (TSA) with and without heme, a physiologically relevant source of Fe, the Δ5 strain grew similarly to its WT parent (Fig. 1A). Supplementation with SN in the presence of heme intoxicated both strains as indicated by the >4 log reduction in colony-forming unit (CFU) when comparing growth on plates containing heme vs heme + SN (Fig. 1A). Using this selective condition, we drop plated *S. aureus* strain Δ5 and incubated plates for 48–72 h. After incubation, we could consistently select for colonies that could resist SN in the Δ5 background (e.g., NJ4 in Fig. 1A). Isolates that displayed stable SN resistance were subjected to whole-genome sequencing and FreeBayes SNP variant analysis. At the outset of our screen, we hypothesized that we might identify novel transporters involved in Fe and/or heme uptake in the Δ5 background. However, of eight isolates tested from two independent experiments, three harbored SNPs in the *perR* gene, suggesting PerR might modulate Fe uptake, contributing to the observed SN resistance. Of the *perR* SNPs, one resulted in a nonsense mutation and two resulted in missense mutations. That the truncated *perR* variant phenocopied the missense variants with respect to streptonigrin resistance suggested to us that PerR would be inactive in each of these mutants. Further repetition of these experiments and screening of nine more isolates via PCR amplification and sequencing of the *perR* gene revealed that seven of the strains harbored SNPs in *perR,* causing missense or nonsense mutations that ostensibly lead to loss of PerR function (Fig. 1B). To determine whether *perR* mutation alone contributes to SN resistance in the presence and absence of hemin, we transduced *perR*::ϕΝΣ from the Nebraska Transposon Insertion Library into the wild-type (WT) USA300-LAC background. This mutant is hereafter referred to as the *S. aureus perR*::Tn strain. That this mutant behaved like a canonical *perR* mutant was confirmed through disc diffusion assays measuring H_2_O_2_ sensitivity, which showed that the *perR*::Tn strain is more resistant to H_2_O_2_ as compared to WT, consistent with previous findings (Fig. S1A and reference 22). In the presence of SN, the *perR*::Tn strain is more resistant to SN killing as compared to WT, irrespective of hemin supplementation (Fig. S1B). Furthermore, provision of *perR in trans* sensitizes the *perR*::Tn strain to both H_2_O_2_ and SN, indicating the observed phenotypes are due to loss of PerR function (Fig. S1A and B).

### The *S. aureus perR::Tn* strain exhibits a growth defect during Fe restriction

**Fig 2:**
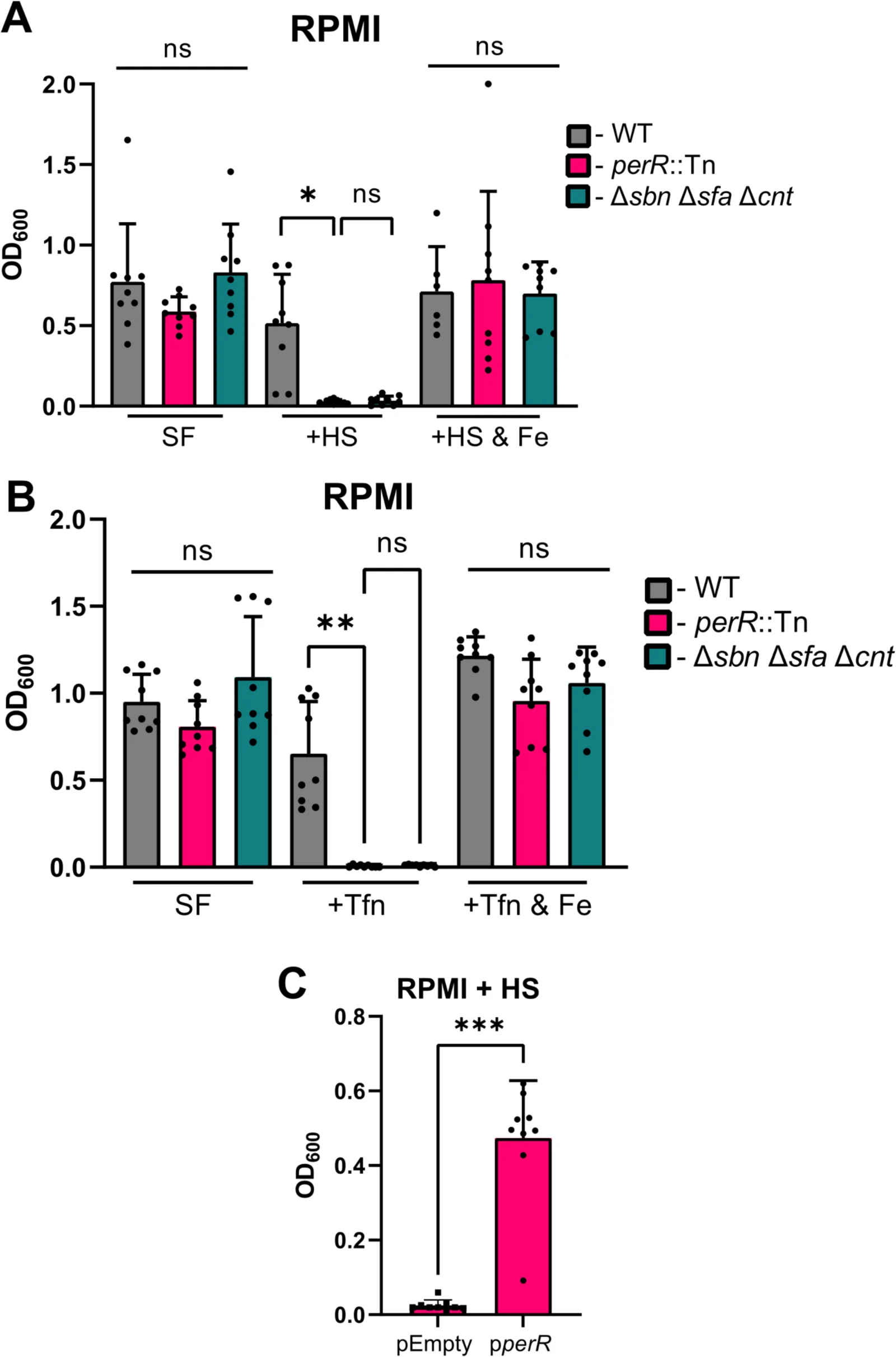
*S. aureus perR::Tn* exhibits a growth defect under conditions of Fe restriction. (**A**) 24-h growth of WT, *perR* mutant (*perR*::Tn), and *sbn*/*sfa*/*cntKLM* mutant *S. aureus* USA300-LAC in RPMI medium containing either no additives (SF), 0.5% vol/vol horse serum (+HS), or 0.5% vol/vol horse serum and 10 μM FeCl_3_ (+HS and Fe). (**B**) 24-h growth of WT *perR* mutant (*perR*::Tn), and *sbn*/*sfa*/*cntKLM* mutant *S. aureus* USA300-LAC in RPMI containing no additives (SF), 50 nM purified human transferrin (Tfn), or 50 nM purified human transferrin and 10 μM FeCl_3_ (+Tfn and Fe). (**C**) 24-h growth of *perR* mutant *S. aureus* USA300-LAC harboring either empty vector (pEmpty) or pALC2073 containing the *perR* gene (p*perR*) in RPMI with 0.5% vol/vol horse serum. In A, B, and C, the bars represent the mean OD_600 nm_ reading ± standard deviation, where each data point represents an individual biological replicate. The data were derived from three independent experiments, and statistical significance was determined using Brown–Forsythe and Welch’s ANOVA with Dunnett’s T3 multiple comparisons test for A and B, and unpaired t-test with Welch’s correction for C. ns indicates no significance; *P < 0.05, **P < 0.01, ****P < 0.0001.

Given that SN activity and Fe are intimately linked, we next explored whether *perR* modulates *S. aureus* growth under conditions of Fe limitation. To this end, we assessed the ability of the *perR*::Tn strain to grow in the chemically defined medium Roswell Park Memorial Institute (RPMI) 1640 with or without supplementation with horse serum (HS), which imparts Fe restriction. In serum-free RPMI, each of the strains WT (USA300), *perR*::Tn, and the *Δsbn Δsfa Δcnt* triple mutant grew similarly. In contrast, in medium containing 0.5% vol/vol HS, WT grew, whereas growth of strains *Δsbn Δsfa Δcnt* and *perR*::Tn was inhibited. Importantly, Fe supplementation rescued both the *perR*::Tn and the *Δsbn Δsfa Δcnt* mutant strains, whereas *perR in trans* rescued the *perR*::Tn strain, indicating the observed growth defect is *perR* and Fe-dependent (Fig. 2A and C). The same pattern of growth was observed when purified human apo-transferrin was used in place of HS, indicating host-derived Fe-binding proteins can impair growth of the *perR*::Tn strain (Fig. 2B).

### SA- and SB-mediated Fe acquisition are defective in a *S. aureus perR* mutant

**Fig 3:**
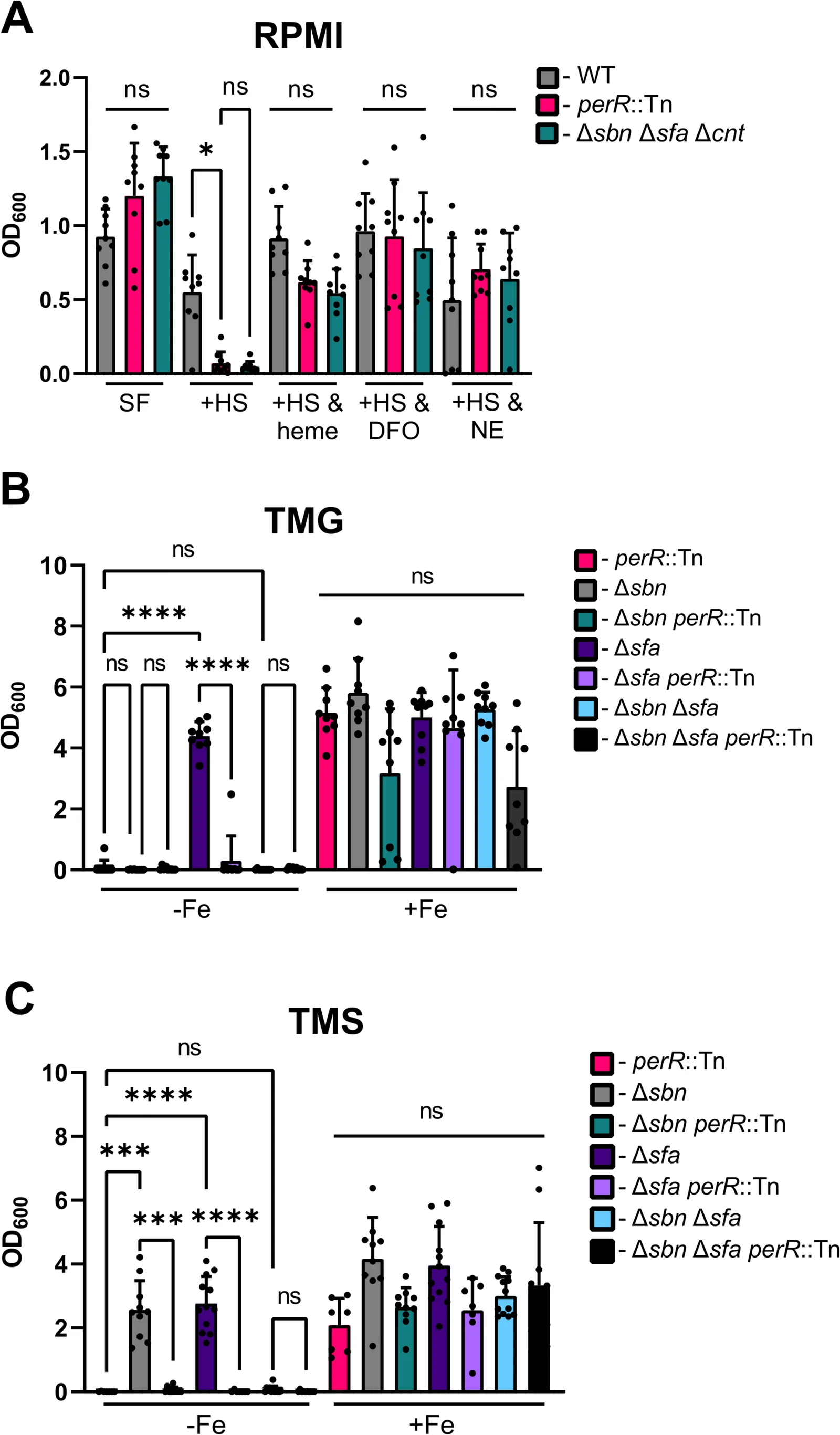
Phenotypic evaluation of known Fe acquisition systems in a *perR* mutant. (**A**) 24-h growth of wild-type, *perR* mutant (*perR*::Tn), and *sbn*/*sfa*/*cntKLM* mutant *S. aureus* USA300-LAC in RPMI medium containing either no additives (SF), 0.5% vol/vol horse serum (+HS), 0.5% vol/vol horse serum and 50 nM bovine hemin (+HS & heme), 0.5% vol/vol horse serum, and 100 μM desferroxamine mesylate (+HS and DFO), or 0.5% vol/vol horse serum and 100 μM norepinephrine (+HS and NE). (**B**) 24-h growth of *S. aureus* USA300-LAC harboring mutations in *perR* (*perR*::Tn), *sbn*, or *sfa* in Fe-restricted TMG medium containing either no additives (−Fe) or 10 μM FeCl_3_ (+Fe). (**C**) 24-h growth of *S. aureus* USA300-LAC harboring mutations in *perR* (*perR*::Tn), *sbn*, or *sfa* in Fe-restricted Tris-minimal succinate (TMS) medium containing either no additives (−Fe) or 10 μM FeCl_3_ (+Fe). In A, B, and C, the bars represent the mean OD_600 nm_ reading ± standard deviation, where each data point represents an individual biological replicate. The data were derived from three independent experiments, and statistical significance was determined using Brown–Forsythe and Welch’s ANOVA with Dunnett’s T3 multiple comparisons test. ns indicates no significance; *P < 0.05, ***P < 0.001, ****P < 0.0001.

The *perR*::Tn strain failed to grow when restricted for Fe, prompting us to explore this phenotype further and pivot away from investigating the mechanism of resistance to SN; we discuss possible mechanisms of SN resistance in the Discussion. We next decided to investigate whether the significant growth defect of the *perR*::Tn strain in Fe-restricted media was due to defects in siderophore production and/or utilization. To this end, we first tested whether well-characterized Fe sources, taken up through specific Fe-acquisition systems, could rescue the growth of the *perR*::Tn strain. Here, we found supplementation of RPMI-HS with hemin (utilized through the Isd pathway), desferroxamine (taken up by the Fhu system), or norepinephrine (taken up by the Sst system), rescued growth, indicating these Fe acquisition systems are functional in the *perR*::Tn strain (Fig. 3A). We next assessed the functionality of the endogenous siderophores SA and SB. Previously, we have shown that growth under Fe restriction is SB-dependent in the presence of glucose due to carbon catabolite repression of TCA cycle activity, which is required for SA synthesis (17). As such, we used Chelex-100-treated Tris-minimal glucose (TMG) medium and evaluated the growth of the *perR*::Tn strain. Using this medium, we found that SB-deficient *S. aureus* failed to grow whereas SA-deficient *S. aureus*, which is competent for SB production, displayed robust growth (Fig. 3B). Remarkably, growth of the *perR*::Tn strain, like the Δ*sbn* mutant strain, was ablated suggesting this strain fails to make and/or use SB (Fig. 3B). Importantly, growth of each strain was rescued by supplementation with FeCl_3_ , indicating the observed defect was due to impaired high-affinity Fe acquisition (Fig. 3B). We also assessed the growth of these mutants in Chelex-100-treated Tris minimal medium containing succinate (TMS), in which *S. aureus* can produce both SA and SB (17). Using TMS, we observed that *S. aureus* lacking the ability to make either SB or SA alone was able to grow, whereas the strain lacking SB and SA production was completely inhibited (Fig. 3C). In this medium, inactivation of *perR* alone or in either of the siderophore deficient mutants rendered *S. aureus* unable to grow unless FeCl_3_ was provided exogenously (Fig. 3C). Taken together, these data indicate that *perR* inactivation compromises the ability of *S. aureus* to grow in an SA- and SB-dependent manner.

### The *S. aureus perR*::Tn strain fails to produce SA and SB to support growth

**Fig 4:**
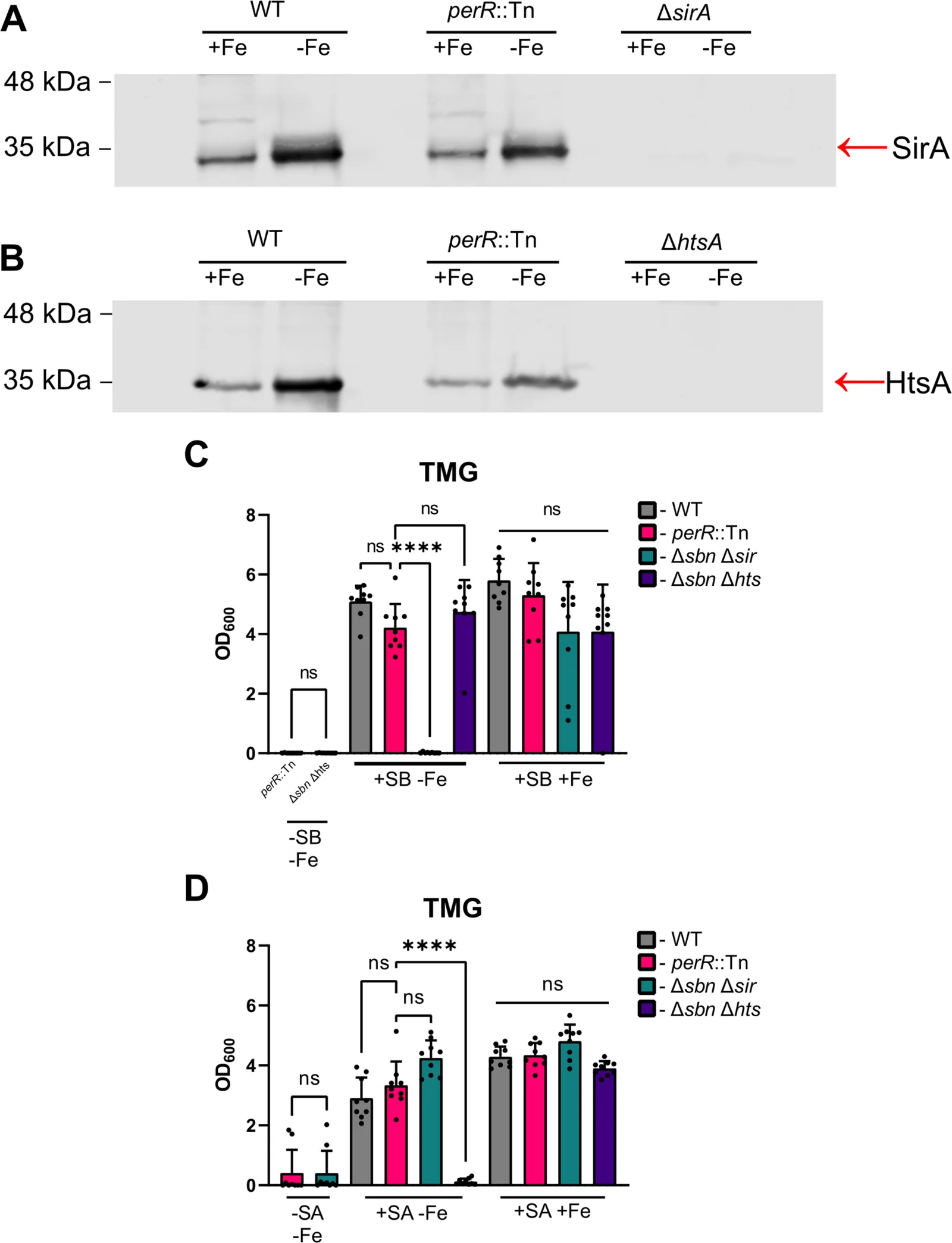
*perR* mutants express endogenous siderophore transporters and utilize SA and SB. (**A**) Western blot of whole-cell lysates of WT USA300, USA300 *perR*::Tn, and Δ*sirA S. aureus* against SirA. (**B**) Western blot of whole-cell lysates of WT USA300, USA300 *perR*::Tn, and Δ*htsA S. aureus* against HtsA. Proteins were extracted from strains grown overnight at 37°C with shaking in Fe-restricted TMG with no additives (−Fe) or supplemented with 10 µM FeCl_3_ (+Fe). (**C**) 24-h growth of *S. aureus* WT, *perR*::Tn, Δ*sbn* Δ*sir*, and Δ*sbn* Δ*hts* mutants in Fe-restricted TMG with supplemented staphyloferrin B-containing supernatant. USA300 *perR*::Tn (leftmost) and Δ*sbn* Δ*hts* were both grown in Fe-restricted TMG with no additives to validate supernatant-mediated growth rescue. (**D**) 24-h growth of WT, USA300 *perR*::Tn, Δ*sbn* Δ*sir*, and Δ*sbn* Δ*hts S. aureus* in Fe-restricted TMG with supplemented staphyloferrin A-containing supernatant. USA300 *perR*::Tn (leftmost) and Δ*sbn* Δ*sir* were both grown in Fe-restricted TMG with no additives to validate supernatant-mediated growth rescue. *Δsbn* strains cannot produce staphyloferrin B, Δ*sir* strains cannot utilize staphyloferrin B, and Δ*hts* strains cannot utilize staphyloferrin A. SB = 10% (vol/vol) staphyloferrin B-containing supernatant obtained as outlined in methods, SA = 10% (vol/vol) staphyloferrin A-containing supernatant obtained as outlined in methods, Fe = 10 µM FeCl_3_. In C and D, the bars represent the mean OD_600 nm_ reading ± standard deviation, where each data point represents an individual biological replicate. The data were derived from three independent experiments, and statistical significance was determined using Brown–Forsythe and Welch’s ANOVA with multiple comparisons. ns indicates no significance; ****P < 0.0001.

Given our data indicate that the *perR*::Tn strain displays defects in SA- and SB-mediated Fe acquisition, we sought to explore the mechanism that underpins this phenomenon. As a first step toward this goal, we investigated whether the *perR*::Tn strain expresses SirA and HtsA, the receptors required for SB and SA utilization, respectively. Western blot analysis revealed that, similar to WT, the *perR*::Tn strain expressed both SirA and HtsA, and expression was influenced by Fe supplementation (Fig. 4A and B). Next, we investigated whether the *perR*::Tn strain could utilize SB or SA if each were provided exogenously. To this end, we provided sterile culture supernatant derived from *S. aureus* lacking the *sfa* locus, which can only produce SB, or lacking the *sbn* locus which can only produce SA, as a source of siderophore. Using SB-containing supernatant, we found that growth of the *perR*::Tn strain in chelexed TMG could be restored to WT levels (Fig. 4C). That SB was the factor promoting growth was established by the observation that a control strain, the Δ*sbn* Δ*sir* strain, which cannot make or utilize SB, was unable to grow with added supernatant unless FeCl_3_ was provided (Fig. 4C). We performed a similar experiment using sterile culture supernatant containing only SA and observed a similar pattern of growth for strain *perR*::Tn, where SA-containing supernatant rescued growth of the *perR*::Tn strain. Using a Δ*sbn* Δ*hts* mutant that cannot synthesize SB or use SA, we showed that SA was the growth-promoting factor in the provided supernatant as this control strain failed to grow (Fig. 4D). Taken together, these data reveal that the *perR*::Tn strain fails to grow under Fe-restricted conditions, and this growth can be rescued by providing SA and/or SB, which might suggest an inability to produce these siderophores itself.

### *S. aureus perR*::Tn exhibits decreased expression of siderophore biosynthetic genes

**Fig 5:**
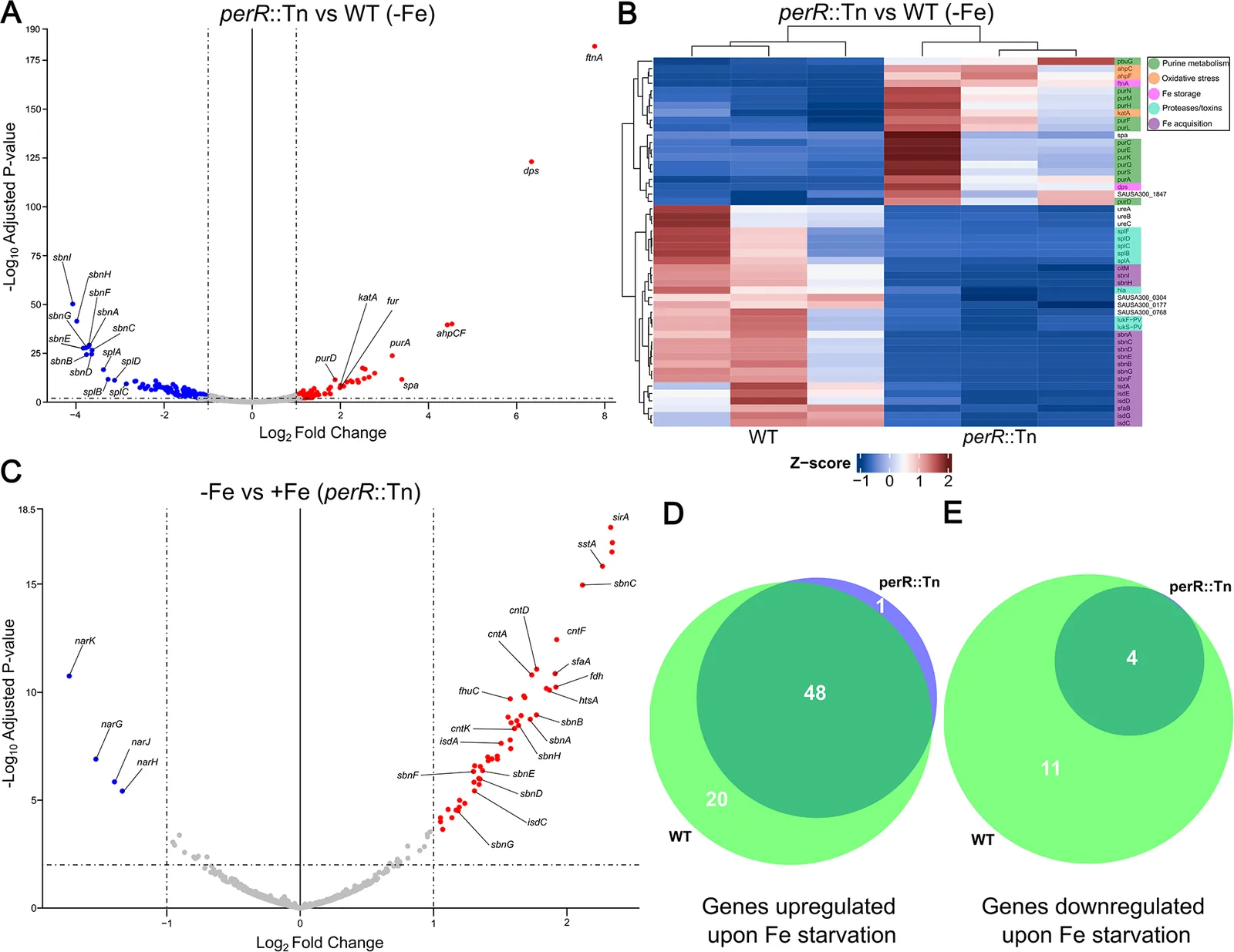
Bulk RNA-seq of *S. aureus* WT and *S. aureus perR* under Fe-restricted and replete conditions. Cells were grown to early exponential phase in TSB containing EDDHA as described in methods and subject to addition (Fe-replete) or no addition (Fe-restricted) of FeCl_3_. (**A**) Volcano plot of differentially expressed genes in *perR*::Tn relative to wild-type *S. aureus* USA300-LAC under conditions of Fe restriction. Horizontal dotted line represents p-value cutoff of 0.01; vertical dotted lines represent fold change cutoff of 2 in either direction. Red points represent genes with significantly increased expression, blue genes with significantly decreased expression, and gray genes below one or both cutoffs. (**B**) Heatmap depicting z-score normalized transcripts per million (TPM) for the top 50 most differentially expressed genes by *P*-value in *perR*::Tn relative to WT under conditions of Fe restriction. (**C**) Volcano plot of differentially expressed genes in Fe-restricted cells relative to Fe-replete cells of *perR*::Tn. Horizontal dotted line represents *P*-value cutoff of 0.01; vertical dotted lines represent fold change cutoff of 2 in either direction. Red points represent genes with significantly increased expression, blue genes with significantly decreased expression, and gray genes below one or both cutoffs. (**D**) Comparison of the number of significantly upregulated genes in Fe-restricted media by WT (green) or *perR*::Tn (blue) *S. aureus* USA300-LAC. (**E**) Comparison of the number of significantly downregulated genes in Fe-restricted media by WT (green) or *perR*::Tn (blue) *S. aureus* USA300-LAC. Lists of significantly upregulated and downregulated genes under all conditions can be found in Table S1.

Given PerR is a transcriptional regulator, we sought to obtain a global picture of gene expression in the *perR*::Tn strain under Fe-restricted conditions. To this end, we performed bulk RNA-seq on the WT and *perR*::Tn strains grown in Fe-restricted (TSB containing EDDHA) and -replete (TSB containing EDDHA and Fe) conditions. Under conditions of Fe restriction, the *perR*::Tn strain, as compared to WT, displayed increased expression of genes previously reported to be repressed by PerR in *S. aureus* and other gram-positive bacteria (23, 30). This included *ftnA* (log_2_ fold change = 7.8), *dps* (6.3), *ahpCF* (4.4 to 4.5), *katA* (2.0), and *fur* (2.0) (Fig. 5A and B and Table S1). Strikingly, the genes most significantly downregulated in the *perR*::Tn strain, relative to WT, comprise the *sbn* operon (-4.1 to −3.8) responsible for SB biosynthesis. Of note, transcripts from the *sfaABC* operon were also decreased (−1.9). The decreased transcription of both these operons was confirmed via RT-qPCR (Fig. S3C and D). We also observed altered expression of genes related to purine metabolism such as *purEKCSQLFMNHD* (1.9–2.8), *purA* (3.2), *guaC* (1.7), and *pbuG* (2.1), as well as altered expression of genes encoding virulence factors such as *spa* (3.4), *hla* (−2.2), *lukFS* (−2.1), *plc* (−1.9), *nuc* (−1.2), and proteases such as the *spl* genes (−2.4 to −3.4), *scpA* (−1.4), and *sspABC* (-2.3 to −2.5) (Fig. 5A and B), indicating *perR* activity can influence the expression of an array of genes reported to impact bacterial fitness during infection. Differences in the expression of some of these genes between the WT and *perR*::Tn strains were also observed in Fe-replete conditions; however, to a lesser extent than under Fe restriction (Fig. S3A). Next, we sought to determine whether the transcriptional response to Fe limitation differed between the WT and *perR*::Tn strains. When comparing the transcriptome of each strain between Fe-restricted and Fe-replete conditions, we observed considerable overlap in the genes upregulated and downregulated between WT and *perR*::Tn strains (Fig. 5D and E). In all, 48 of the 68 genes upregulated upon Fe restriction in WT were also upregulated in the *perR*::Tn strain; these were comprised of many Fur-regulated Fe acquisition loci, including *isd*, *cnt*, *sfa*, *sbn*, *fhu*, *sst*, *sir*, and *hts* (Fig. 5C). Only 4 genes of the 15 downregulated by WT upon Fe restriction were shared with the *perR*::Tn strain; these comprised the *narGHJK* operon. Of the 11 exclusively downregulated by WT and not the *perR*::Tn strain upon Fe restriction, roughly half were comprised of known PerR regulon members (*katA*, *ftn*, *dps*, and *ahpCF*) (Fig. S3B). Taken together, our analyses suggest that the *perR*::Tn strain, for the most part, responds to Fe restriction at the transcriptional level similarly to WT *S. aureus*. Despite this, the *perR*::Tn strain has greatly reduced transcripts from the *sbn* and *sfa* loci, which presumably impairs siderophore biosynthesis and growth under Fe restriction.

### Inactivation of *fur* restores *S. aureus perR*::Tn growth in an *sbn*-dependent manner

**Fig 6:**
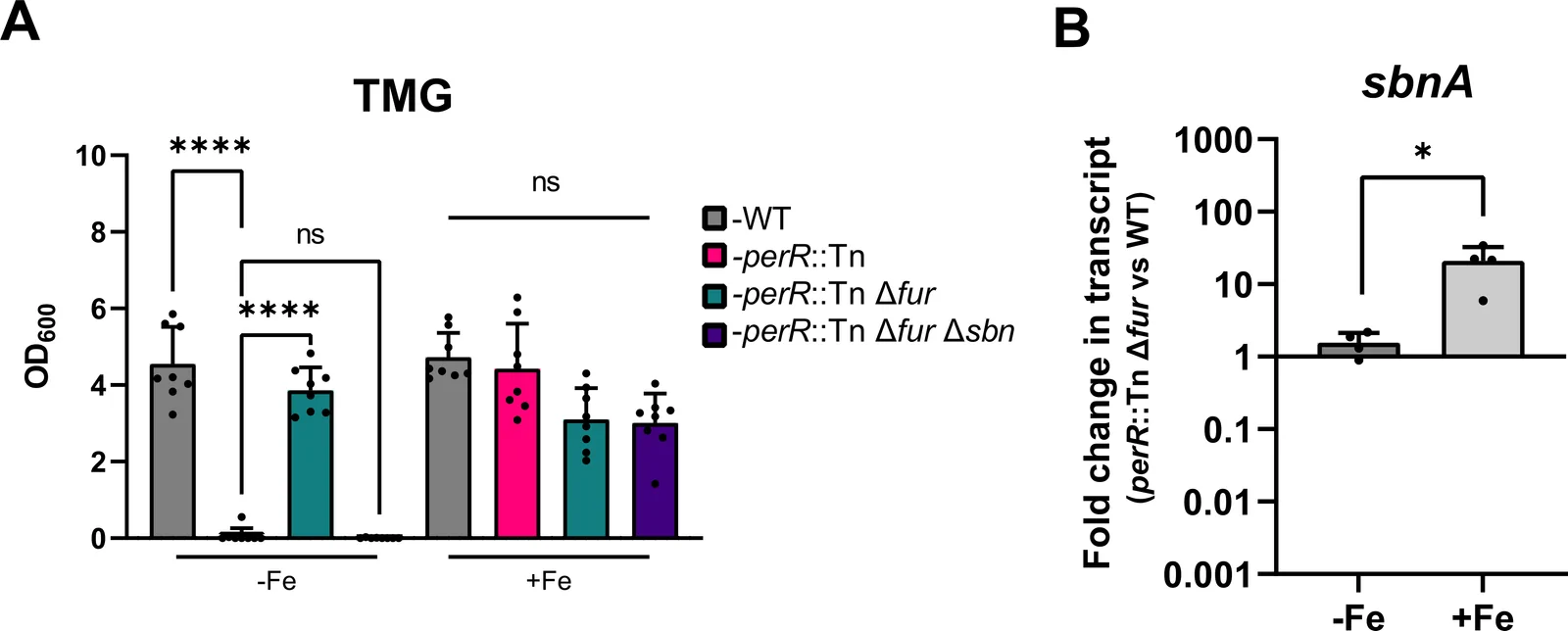
Mutation of *fur* in a *perR* mutant background rescues growth in an *sbn*-dependent manner. (**A**) 24-h growth of *S. aureus* USA300-LAC harboring mutations in *perR* (*perR*::Tn), *fur*, and *sbn* in Fe-restricted TMG medium containing either no additives (−Fe) or 10 μM FeCl_3_ (+Fe). (**B**) Fold change in *sbnA* transcript of *perR*::Tn Δ*fur* relative to WT *S. aureus* USA300-LAC as measured through RT-qPCR and calculated via the ΔΔC_T_ method. −Fe = Fe-restricted conditions, +Fe = Fe-replete conditions. Cells were grown to early exponential phase in TSB containing 2,2-dipyridyl as described in methods and subject to the addition of ferric chloride (Fe-replete) or no intervention (Fe-restricted). In A, the bars represent the mean OD_600 nm_ reading ± standard deviation, where each data point represents an individual biological replicate. The data were derived from three independent experiments, and statistical significance was determined using Brown–Forsythe and Welch’s ANOVA with multiple comparisons. In B, the bars represent the mean fold change in transcript as determined via ΔΔC_T_ method ± standard deviation, where each data point represents an individual biological replicate. The data were derived from two independent experiments, and statistical significance was determined using an unpaired t-test with Welch’s correction. ns indicates no significance; *P < 0.05, ****P < 0.0001.

Our observation that *sbn* transcription is greatly reduced in *S. aureus perR*::Tn relative to the WT, even in Fe-restricted media, led us to speculate that this could underpin the observed *perR*-dependent growth defect. That *sbn* expression in the *perR*::Tn strain was influenced by Fe also indicated that Fur regulation was intact. As Fur acts as a Fe-responsive repressor protein for the *sbn* and *sfa* loci, we reasoned that inactivation of *fur* might increase *sbn* and *sfa* transcription and rescue growth of the *perR*::Tn strain under Fe restriction (13, 27). Remarkably, inactivation of *fur* in the *perR*::Tn strain restored growth in chelexed TMG, and mutagenesis of *sbn* in the *perR*::Tn Δ*fur* strain background ablated this growth, indicating SB synthesis was responsible for this observation (Fig. 6A). RT-qPCR analysis of *sbn* transcript levels in the *perR*::Tn Δ*fur* strain confirmed that transcription of the operon was restored to near WT levels in Fe-restricted conditions, and transcription was greatly increased relative to WT even in Fe-replete conditions (Fig. 6B). Repetition of this experiment in succinate-containing medium to evaluate SA-mediated Fe acquisition revealed that strains with *fur* mutation were unable to grow, regardless of Fe availability, indicating factors in addition to Fe were influencing bacterial growth under this condition (Fig. S5A).

Given the impact of *fur* inactivation on SB-dependent growth of the *perR*::Tn strain*,* we next sought to determine whether PerR was directly interacting with the *sbn* promoter. We identified two potential PerR-binding sites in the promoter region of *sbnA*, suggesting PerR might directly regulate *sbn* operon expression by DNA binding (Fig. S4A). Electrophoretic mobility shift assays (EMSAs) were performed, and while they demonstrate that PerR bound to the canonical PerR-regulated promoter/operator sequence of *ahpC,* which was used as a positive control, we were unable to demonstrate binding of PerR to the *sbnA* promoter/operator sequence (Fig. S4B).

### The *S. aureus perR*::Tn strain displays decreased virulence in a murine skin infection model

**Fig 7:**
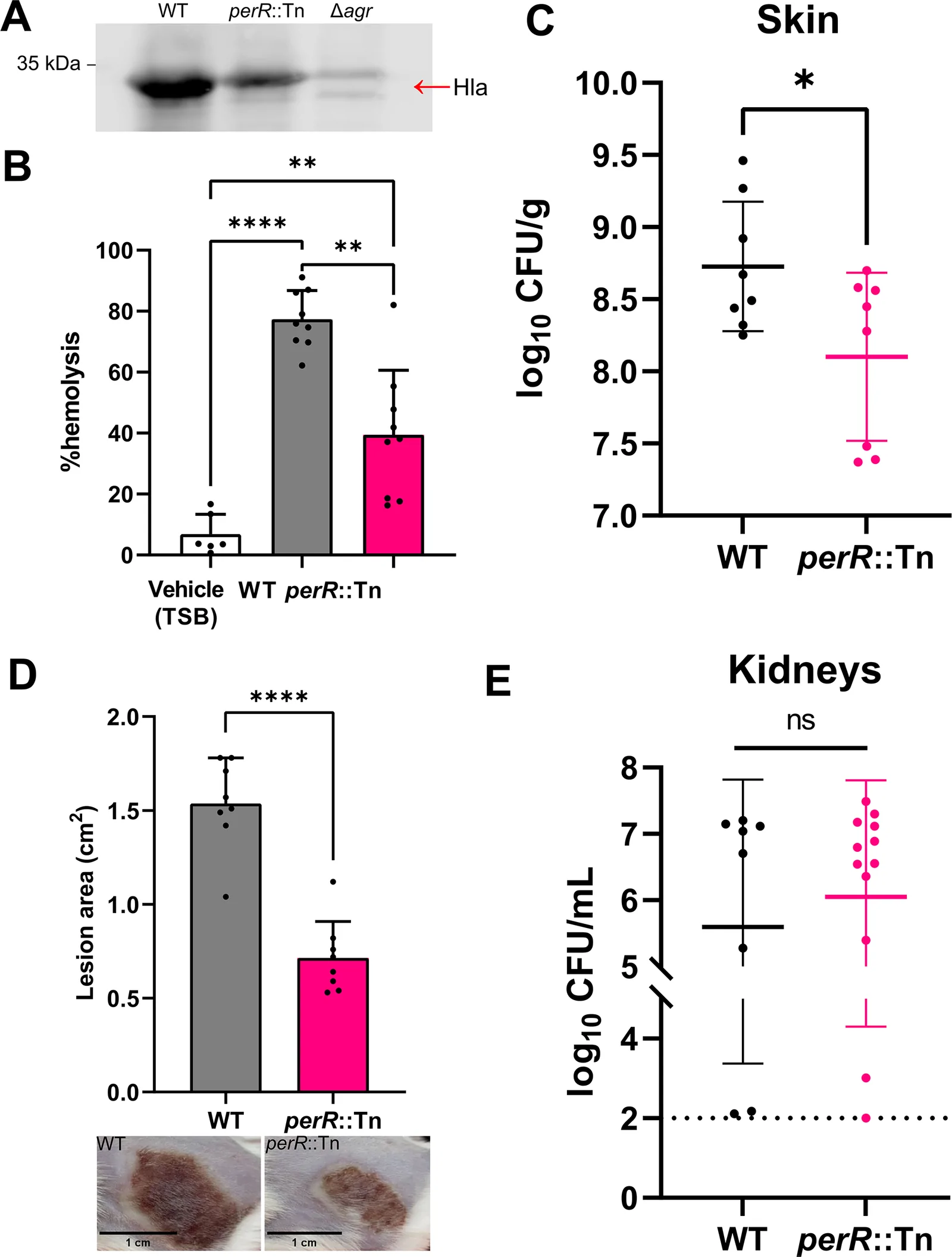
*perR* mutants exhibit decreased virulence in skin abscesses. (**A**) Western blot of secreted culture supernatant against alpha-hemolysin. (**B**) Hemolytic capabilities of *S. aureus* perR::Tn culture supernatant on defibrinated sheep’s blood. Bars represent the mean % hemolysis ± standard deviation, where each data point represents an individual biological replicate. The data were derived from three independent experiments, and statistical significance was determined using Brown–Forsythe and Welch’s ANOVA with Dunnett’s T3 multiple comparisons test. (**C**) Bacterial burden within skin lesions. Mice were injected subcutaneously with an initial dose of ~4.0E7 CFU on each flank. The line represents the mean log_10_ CFU/g recovered from lesion tissue ± standard deviation where each data point represents an individual mouse. (**D**) Mean area of dermonecrotic lesions formed at 48 hpi from the same infection with representative images from mice injected with either *S. aureus* strain. Bars represent mean lesion area ± standard deviation, where each data point represents an individual biological replicate. The data were derived from two independent experiments and statistical significance was determined using an unpaired t-test with Welch’s correction. (**E**) Bacterial burden within the murine kidney following 96-h systemic infection with either WT or perR::Tn USA300-LAC. Mice were injected with initial dose of ~6.0E6-1.0E7 CFU via tail vein. Line represents the mean log10 CFU/mL recovered from organ homogenate ± standard deviation where each data point represents an individual mouse. The data were derived from two independent experiments and statistical significance was determined using an unpaired t-test with Welch’s correction. ns indicates no significance; *P < 0.05, **P < 0.01, ****P < 0.0001.

Our RNA-seq data revealed that the *perR*::Tn strain displayed altered expression of genes known to contribute to *S. aureus* infection (31–35). Indeed, in addition to siderophore biosynthesis genes, proteases and toxins such as alpha-hemolysin (Hla) were downregulated as compared to WT (Fig. 5B and Table S1). Western blot analysis of TCA precipitated culture supernatants from WT, *perR*::Tn, and *agr* null strains revealed that the *perR*::Tn strain expresses ~50% of WT Hla, whereas the *agr* mutant produces almost none (Fig. 7A). In accordance with these observations, we observed that sterile culture supernatants from the *perR*::Tn strain were also less hemolytic toward defibrinated sheep’s blood (Fig. 7B). Given that the formation of dermonecrotic lesions in the murine skin is driven by Hla (31, 36), we next performed murine skin infections to evaluate the impact of PerR inactivation on lesion formation and bacterial burden. Here, we observed a decreased bacterial burden for the *perR*::Tn strain as compared to WT at 48 hpi (Fig. 7C). Furthermore, *S. aureus perR*::Tn-infected mice formed smaller lesions relative to WT-infected mice, indicating that, in this model, the *perR*::Tn strain is less pathogenic (Fig. 7D). We also explored whether the *perR*::Tn strain was attenuated in a systemic model of murine infection. Here, we infected mice with WT and *perR*::Tn strains and assessed the bacterial burden in the murine kidney at 96 hpi. Despite global changes in gene expression attributed to loss of *perR,* we found that the burden of *S. aureus* in the kidney was not affected by *perR* inactivation (Fig. 7E).

### The *perR* gene is highly conserved in clinical isolates of *S. aureus*

**Fig 8:**
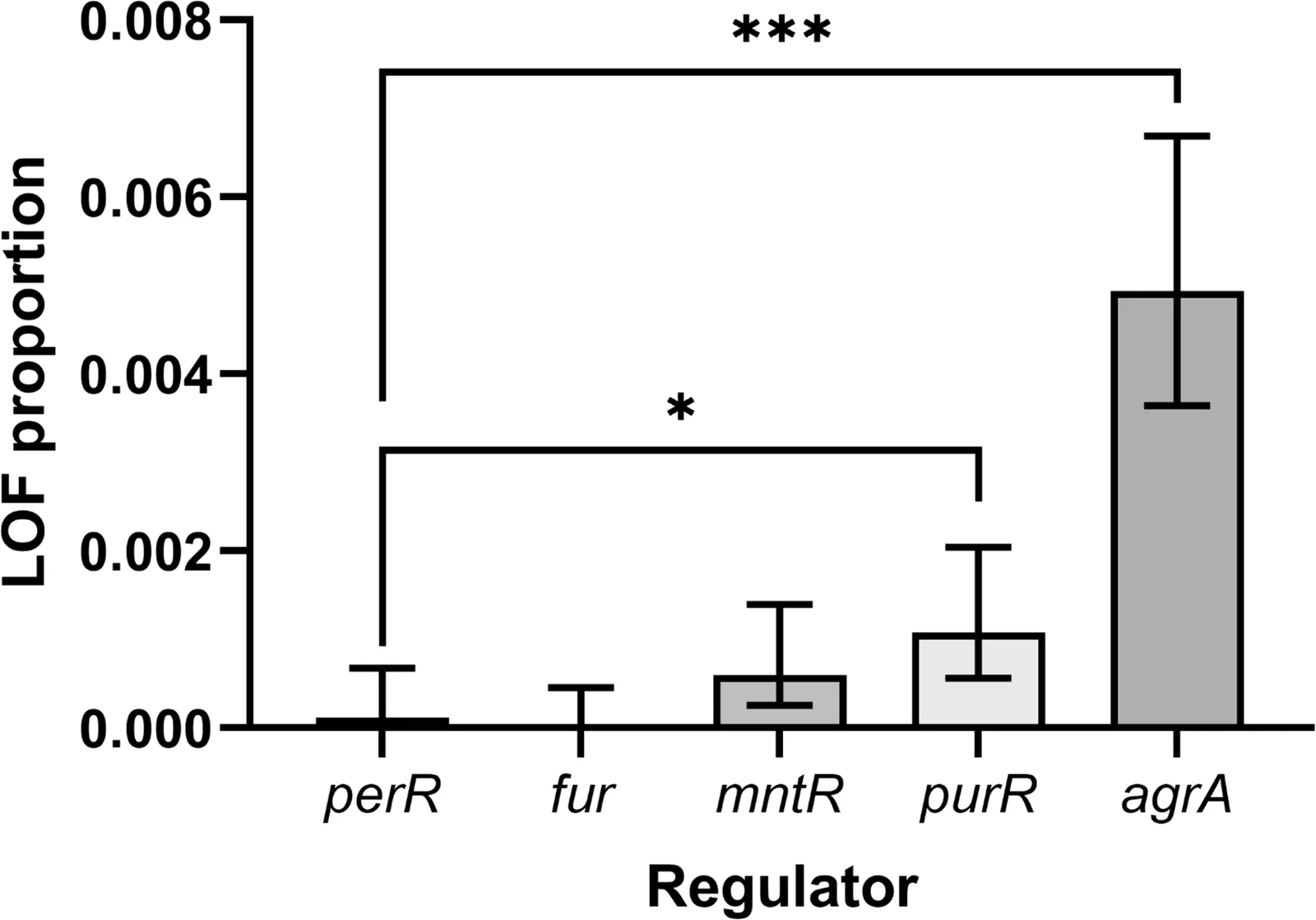
Conservation of *perR* relative to other transcriptional regulators. LOF frequency was first assessed in *perR* and subsequently compared with additional regulators (*fur*, *mntR*, *purR*, and *agrA*) across a collection of clinical *Staphylococcus aureus* isolates. Bars indicate the proportion of isolates harboring predicted LOF variants; error bars denote 95% Wilson confidence intervals. Statistical significance relative to *perR* was assessed using Fisher’s exact test with Benjamini–Hochberg correction for multiple testing (*q* < 0.05). Non-significant comparisons not shown; *P < 0.05, ***P < 0.001.

The importance of PerR to Fe-restricted growth led us to speculate on its importance in human infection. We examined the presence of predicted loss-of-function (LOF) variants due to frameshift or nonsense mutations in the *perR* gene across a collection of >8,000 *S*. *aureus* human bloodstream infection isolates. LOF variants in *perR* were rare, indicating strong conservation in the population. To assess whether the conservation of *perR* was regulator-specific, we extended the analysis to additional transcriptional regulators (i.e., *fur*, *mntR*, *purR*, and *agrA*). The *fur* gene also showed low LOF frequency and did not differ significantly from *perR*. In contrast, *purR* exhibited modest but significant enrichment of LOF variants, while *agrA* showed a pronounced increase in LOF frequency. These differences were confirmed by Fisher’s exact test with false discovery rate correction, indicating differential selective constraint across regulators and supporting strong conservation of *perR* and *fur* (Fig. 8).

## DISCUSSION

In this work, we show that a *S. aureus perR*::Tn strain displays altered Fe homeostasis. Indeed, *perR*-deficient *S. aureus* exhibits Fe-dependent growth defects in Fe-restricted media which can be restored by *perR* complementation. Through manipulation of culture conditions by providing different Fe and carbon sources, we have established that the *perR*::Tn strain is deficient in SB-mediated Fe acquisition owing to significantly decreased transcription of the *sbn* genes that are required for SB biosynthesis. Similarly, we observed reduced transcription of the *sfa* locus, required for SA biosynthesis, and observed SA-dependent growth defects under Fe restriction. Together, our data show that PerR helps coordinate expression of siderophore biosynthesis genes in *S. aureus* and is required for staphyloferrin-dependent growth.

**Fig 9:**
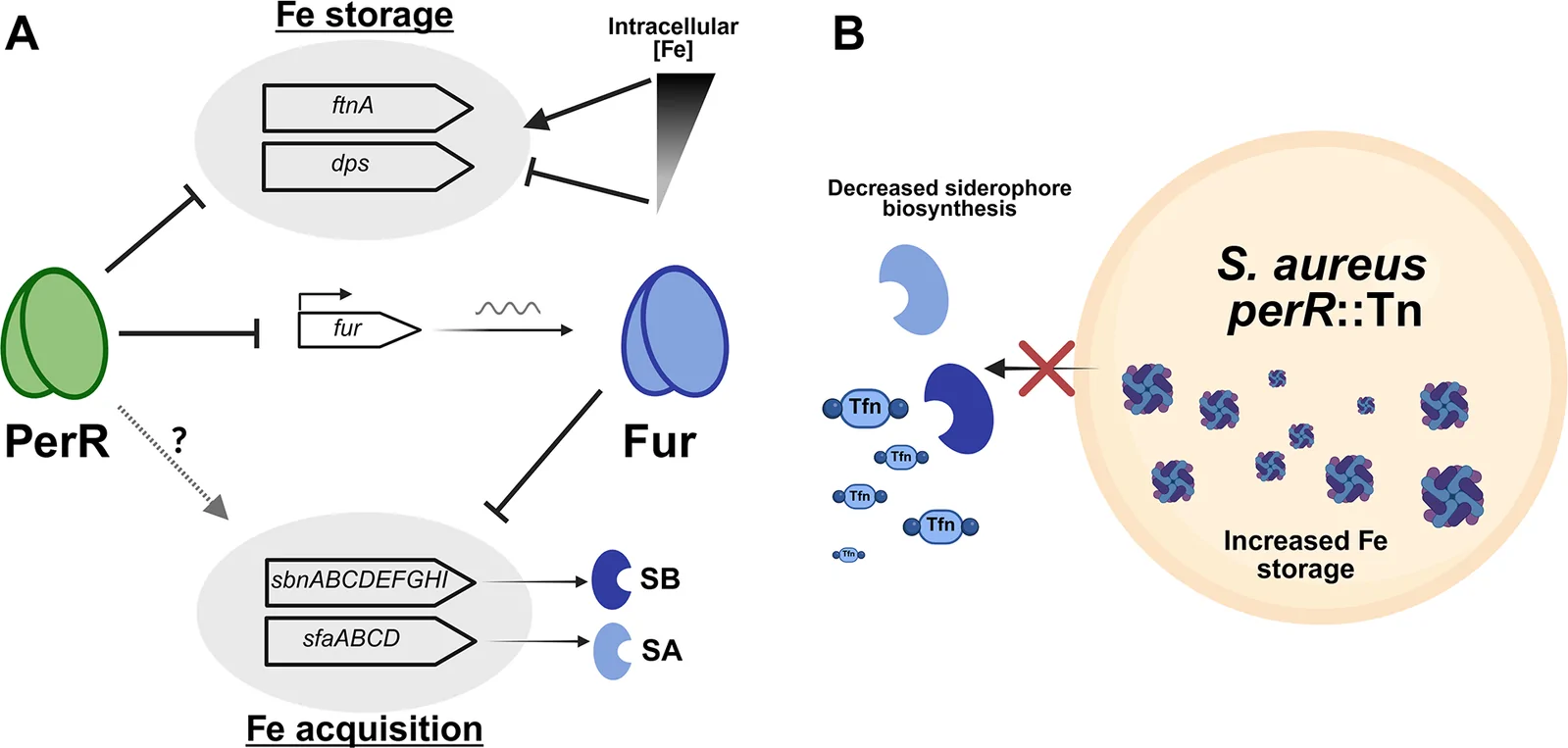
Simplified model of the role of PerR and Fur in *S. aureus* iron homeostasis. (**A**) The influence of PerR and Fur in iron homeostasis in *S. aureus*, and (**B**) the possible implications of *perR* mutation on resistance to streptonigrin. Figure created in BioRender with license (Sheikh, A. [2026] https://BioRender.com/nctis8p).

*S. aureus perR* mutants were isolated through selection for SN resistance. The activity of SN requires intracellular Fe and aerobic conditions (37, 38); however, the mechanism of SN toxicity does not involve diffusible ROS intermediates, and bacterial ROS-detoxifying enzymes play no role in resistance (28). As reported in the literature and confirmed here in our RNA-seq data, the *perR*::Tn strain expressed elevated transcripts for *ftnA* and *dps*, which code for Fe storage proteins (23). Previous literature has shown that *S. aureus* lacking *ftnA* displays heightened sensitivity to SN (39). Consistent with this observation, increased storage and sequestration of intracellular Fe alone because of *perR* inactivation might better protect *S. aureus* from SN toxicity (Fig. 9). Indeed, in the Δ5 background devoid of siderophore, where we initially identified *perR* as a factor affecting intracellular Fe, this could conceivably have been the case. To test this directly, the mutation of *ftnA* or *dps* in the *perR* background and challenge with SN under the conditions described in our screen could be performed. Regardless, *perR* mutation has pleiotropic effects, and ostensibly SN resistance in the *perR*::Tn strain is due to concerted changes that also include defective siderophore-dependent Fe uptake (Fig. 9). Indeed, siderophore mutants of *S. aureus* can display increased resistance to SN (27, 40). While our initial goal was to identify transporters involved in Fe uptake, we instead identified a transcriptional regulator with pleiotropic effects on Fe storage and uptake.

Previous studies of PerR function in *S. aureus* 8325-4 have established crosstalk between the oxidative stress response and Fe restriction response, with co-regulation of catalase (*katA*) and ferritin (*ftnA*) by both PerR and Fur (23, 24, 30). These studies reported that *perR* inactivation slowed *S. aureus* growth in metal-chelated medium, which could be alleviated by Fe supplementation. In those previous studies, it was proposed that growth impairment is as a result of ferritin overexpression (23). In this work, we further characterize and expand upon the defective growth of a *S. aureus perR*::Tn mutant strain, in the USA300-LAC background, and have established that *perR* mutants downregulate Fe acquisition genes in addition to confirming the upregulation of Fe storage proteins as previously demonstrated. Our EMSA data did not reveal direct binding of PerR to the *sbnA* promoter, despite the high degree of similarity between the consensus and putative *sbnA* PerR box sequences. Despite this, it is conceivable that while recombinant PerR could bind the *ahpC* promoter probe, the conditions for PerR binding to the *sbnA* promoter probe were not met *in vitro*. Alternatively, the impact of *perR* inactivation on *sbn* transcription might be indirect. This notion is supported by our growth assays using the *perR*::Tn *fur* and *perR*::Tn *fur sbn* mutants, where *fur* inactivation rescued growth of the *perR*::Tn strain in a *sbn*-dependent manner (see Fig. 6A). This could suggest that the influence over *sbn* transcription is mediated through Fur, which is upregulated in strain *perR*::Tn as shown in our RNA-seq analysis and in previous work (23). What remains unclear, however, is why Fur overexpression, as seen in the *perR*::Tn strain, seems to selectively repress *sbn* transcription so strongly. We also considered a second scenario where this effect was mediated by Fpa. Fpa was recently shown to interact with Fur to alleviate repression of Fur-dependent transcription under Fe-restricted conditions (41). We considered the possibility that, under Fe restriction, a *perR* mutation led to decreased *fpa* expression; however, our RNA-seq data, taken at early- to mid-log-phase growth, did not support this. Nonetheless, that *sbn* transcription is also influenced by heme, and SB biosynthesis is not influenced by carbon catabolite repression, suggesting that SB is a key siderophore for *S. aureus,* and its expression is highly coordinated (17, 21).

Given that the *S. aureus perR*::Tn strain fails to grow in serum and that *perR* has a low frequency of LOF mutations across a large cohort of human bloodstream isolates (see Fig. 8), we hypothesized that the *perR* inactivation would attenuate *S. aureus in vivo* during murine systemic infection. Remarkably, despite such a strong growth defect *in vitro,* we found that *perR* inactivation did not alter the burden of *S. aureus* in the murine kidney. However, as indicated in our growth assays, the *perR*::Tn strain maintains the ability to utilize heme (see Fig. 3A) despite initially being isolated in a screen in which heme utilization was selected against, and within the kidney, heme can be used as a nutrient (42). Furthermore, niche-specific siderophore production may influence the contribution of SA and/or SB to *S. aureus* fitness in distinct tissues (43). Finally, the ability of *S. aureus* to acquire Fe through additional transporters such as Sst may also influence the *in vivo* growth of the *perR*::Tn strain (14).

Recent work has revealed that PerR positively regulates the *lukAB* genes encoding the LukAB leukocidin (44) and thus links the ROS sensing to *S. aureus* toxin production. Here, our data also showed that, under Fe-restricted conditions, PerR was also required for maximum Hla production. Consistent with this, we observed *in vivo* in the murine skin that the *perR*::Tn strain induced smaller dermonecrotic lesions, and it is known that skin lesions can be attributed to Hla activity (36). The decreased bacterial burden for the *perR*::Tn strain in the skin, however, is ostensibly due to the pleiotropic effects of *perR* inactivation on Fe acquisition and virulence factor expression and not due to decreased Hla alone (45). Interestingly, a *perR* mutant in *S. aureus* strain 8325-4, which is cured of phage elements, has also been reported to show a reduced burden during skin infection (23). It has also been reported that *S. aureus* mutants unable to synthesize SA alone are less able to cause skin lesions, indicating additional factors are, in theory, affecting the virulence potential of a *perR* mutant (46).

The Fur protein is a well-established Fe-dependent regulator of gene expression that was first identified in *E. coli* and is conserved in many bacteria, including *S. aureus* (47, 48). In *S. aureus*, it is evident that additional layers of transcriptional regulation exist to finely coordinate Fe homeostasis. These include, for example, the regulatory small RNA *isrR* that directs a Fe-sparing response (22, 49), the Fpa (YlaN) protein that controls Fur binding to DNA (41), and SbnI, a heme-responsive factor affecting *sbn* operon expression (21). Here, our findings add further to the understanding of Fe-regulated gene expression in *S. aureus*, as we reveal a critical role for PerR in siderophore-mediated Fe-restricted growth and bacterial fitness in murine skin infection.

## MATERIALS AND METHODS

### Strains and culture conditions

Bacterial strains and vectors employed in this study are summarized in Table S2. For routine culture and genetic manipulation, *S. aureus* strains were cultured in TSB at 37°C shaken at 200 rpm, or on TSB solidified with 1.5% (wt/v) agar (TSA) at 37°C.

For Fe-restricted growth assays, *S. aureus* was grown in RPMI 1640 (Wisent), TMS, or TMG liquid at 37°C with shaking at 200 rpm. TMS medium was formulated as previously described (50); however, cysteine was used at a final concentration of 44 µg mL^−1^. TMG medium was formulated similarly, but with 0.4% (wt/vol) glucose as the primary carbon source. TMS and TMG were treated with Chelex-100 resin (Bio-Rad) to remove Fe, used according to the manufacturer’s instructions, and were filter-sterilized before use. Where appropriate, the following antibiotics were used: ampicillin (100 µg mL^−1^), chloramphenicol (5 µg mL^−1^), erythromycin (10 µg mL^−1^), kanamycin (50 µg mL^−1^), neomycin (50 µg mL^−1^), or tetracycline (3 µg mL^−1^).

Methodologies for the generation of strains containing the *sbn*::Tc, *sfa*::Km, *fhuG*::Ery, *sirA*::Km, *hts*::Tc, *fur*::Km, and *agr*::Tc mutations have previously been described (13, 27, 40, 51). For this current study, antibiotic resistance-marked mutations were mobilized into USA300-LAC and derivatives using phage transduction using procedures previously described (52). Mobilization of plasmids listed in Table S2 was similarly performed using phage transduction. Phage lysates were generated from the donor strain using phage 80α, recipient strains were infected, and transductants were selected on plates with appropriate antibiotics added in the presence of citrate. Successful transductions were confirmed by plasmid prep and test digest for plasmids, and PCR with the appropriate primers for chromosomal mutations (Table S3).

Deletion of the *cntKLM* and *sstABCD* genes was achieved by homologous recombination using the temperature-sensitive plasmid pKOR1 following the methods described by Bae and Schneewind (53). Further details for this are found in the accompanying supplementary methods. After creation of the *sst*, a previously described *fhuG* mutation (40) was mobilized into this strain to give rise to Δ5 *S. aureus*.

### Streptonigrin-based screening assays

Isolated colonies from Δ5 *S. aureus* grown on TSA were inoculated into RPMI 1640 with the appropriate antibiotics and cultured O/N. Next, cultures were diluted to an optical density at 600 nm (OD_600_) of 0.1 and a 10-fold dilution series through to 10^−5^ was created for each. Next, 10 µL of each dilution for each strain was drop plated onto TSA containing 200 ng mL^−1^ SN and 1 µM bovine hemin, a concentration at which *S. aureus* heme uptake is Isd-independent (10). Plates were left to incubate at 37 °C for 72 h. Colonies that grew in the presence of SN and subsequently displayed stable resistance in the presence of heme were subject to whole-genome sequencing by SeqCenter, LLC. Genomes were assembled to the USA300 reference sequence (NC_007793) and Bayesian SNP calling was performed with the FreeBayes plug-in using the Geneious Prime software package.

### Growth assays

Endpoint growth assays were performed to assess the growth of strains under conditions of Fe restriction. Strains were inoculated from TSA plates into 2 mL of RPMI 1640, non-Chelex-treated TMS, or non-Chelex-treated TMG medium and grown O/N. Next, strains were subcultured at a starting OD_600_ of ~0.0005 into the same medium that was used for precultures with specific amendments as outlined in experimental results and figure legends. In some instances, RPMI was supplemented with horse serum (Sigma-Aldrich) (0.5% vol/vol) or human serum (25% vol/vol) to further induce Fe restriction. To restrict Fe in TMS and TMG, media were treated with Chelex-100 resin (Biorad) according to the manufacturer’s instructions, and divalent cations other than Fe were added back to the media; final concentrations: 100 µM CaCl_2_, 25 µM ZnCl_2_, 1 mM MgCl_2_, and 25 µM MnCl_2_. For control conditions, 10 µM ferric chloride (Fisher) was added to the media to create Fe-replete growth conditions. In some instances, additional additives were used in growth assays, including 50 nM bovine hemin (Sigma), 100 µM desferroxamine mesylate (DFO) (Pfizer), 100 µM norepinephrine (NE) (Sigma), or sterile culture supernatant containing staphyloferrin A or B at a concentration of 10% (vol/vol). Sterile staphyloferrin B-containing culture supernatant for growth assays was generated by culturing *sfa* mutant *S. aureus* in TMG medium without additives for 24 h, centrifuging cultures at 5,000 × *g* for 10 min, and passing the culture supernatant through a 0.2 µm filter (Fisher). The process was repeated with a *sbn* mutant strain grown in TMS to obtain sterile staphyloferrin A-containing supernatant. Growth assays were performed for 24 or 48 h as indicated, and OD_600_ was measured at the indicated timepoint using an EPOCH 2 microplate reader (BioTek).

### Western blot for siderophore transport lipoproteins

To assess SirA and HtsA protein expression, Western blot analysis was performed as previously described (42) with additional details provided in supplementary methods. In brief, membranes were probed using polyclonal rabbit anti-SirA (27) or anti-HtsA (13) primary antibody and detected using a goat anti-rabbit IR800-conjugated secondary antibody (Li-Cor) diluted to 1:20,000 and then imaged on an Odyssey CLx (Li-Cor) IR imager.

### RNA extraction, RNA-seq, and RT-qPCR

To assess gene expression, we extracted RNA from early exponential-phase cells grown in TSB containing 100 µM EDDHA. Overnight precultures grown in this medium were subcultured in technical duplicate at a starting OD_600_ of 0.1 in fresh media and left to grow for 3 h to an OD_600_ of ~1.0. Next, one of each duplicate was spiked with 50 µM ferric chloride and then all tubes left to grow for another hour with shaking. Cells were normalized to an OD_600_ equivalent of 3.0, pelleted, lysed, and RNA extraction performed with Trizol (Invitrogen) according to the manufacturer’s instructions. Extracted RNA was then subject to rigorous DNase treatment (Invitrogen) and phenol-chloroform extraction to inactivate and remove DNase. Samples for RNA-seq were sent for processing at SeqCentre, LLC; read alignments and differential expression analysis were performed using Geneious Prime software.

RNA used for qPCR was extracted using the same method on cells grown in TSB with 400 µM 2,2′-dipyridyl. To generate cDNA libraries, 500 ng of extracted RNA was used with reaction components from a High-Capacity cDNA Reverse Transcription Kit (Invitrogen) according to the manufacturer’s instructions. cDNA of 0.75 μL was then used as template in qPCRs along with forward and reverse primers (Table S2) (500 nM each) and iTaq Universal SYBR Green Supermix (1×) (Bio-Rad), with water to bring the reaction volume up to 15 µL. qPCR was performed in a Rotor-Gene 5000 (Qiagen) with the default three-step protocol, and fold change was calculated with the ΔΔC_T_ method.

### Western blot for secreted alpha-hemolysin

To assess the expression of secreted Hla, Western blot analysis of bacterial culture supernatants was performed. Following overnight culture, wild-type, *perR*::Tn, and Δ*agr* strains were subcultured at a starting OD_600_ of 0.1 in 25 mL TSB with 100 µM EDDHA and incubated at 37°C with shaking for 6 h until they reached the mid-exponential phase. Samples were normalized to an OD_600_ equivalent of 73, and the bacterial cells were pelleted by centrifugation at 5,000 × *g* for 10 min. The resulting supernatant was filter-sterilized and incubated with an equivalent volume of 20% (wt/vol) trichloroacetic acid for 3 h at 4°C. Precipitated proteins were pelleted by centrifugation at 5,000 × *g* for 20 min, and the resulting pellet was washed twice with ice-cold 70% ethanol before suspension in 70 µL 1× Laemmli buffer. Samples were boiled and subjected to SDS-PAGE. Two gels were run simultaneously and loaded identically to perform Western blot and Coomassie staining. Protein transfer and antibody probing were performed as described in supplementary methods except the membrane was incubated with rabbit anti-Hla (Sigma) diluted to 1:1,000 and donkey anti-rabbit IR800-conjugated secondary antibody (Li-Cor) diluted to 1:20,000. Blots were imaged on an Odyssey CLx (Li-Cor) with emission filter set to 800 nm.

### Hemolysis assays

Hemolysis assays were performed as described previously (54). In brief, culture supernatant was obtained from WT or *perR*::Tn strains grown at a starting OD_600_ of 0.1 in TSB with 100 µM EDDHA at 37°C with shaking until all cultures reached an OD_600_ of 4. Next, the bacteria were pelleted, and the resulting supernatant was filtered. Defibrinated sheep’s blood (Cedarlane) was washed, diluted in sterile PBS to 4% vol/vol, and mixed 1:1 in a 96-well plate with either undiluted sterile culture supernatant, a zero-lysis control (PBS), a full lysis control (PBS + 0.1% [vol/vol] Triton X-100), or a vehicle control (TSB). Plates were incubated for 1 h at 37°C without shaking, after which OD_650_ readings were taken in a Synergy HTX plate reader (Bio-TEK). Percent hemolysis was calculated as the difference between the OD_650_ of a given sample and the zero-lysis control divided by the difference between the OD_650_ of the full lysis and zero lysis control.

### Murine systemic infection and subcutaneous skin infection models

All protocols for murine infection were reviewed and approved by the University of Western Ontario’s Animal Use Subcommittee, a subcommittee of the University Council on Animal Care. To evaluate the relative virulence of *S. aureus perR*::Tn in a murine bacteremia model, 6-week-old male and female BALB/c mice were obtained from Charles River Laboratories and housed in microisolator cages. *S. aureus* strains were grown to the mid-exponential phase (OD_600_ of 2.0–2.5) in 25 mL TSB, washed twice with PBS, and resuspended in PBS to an OD_600_ of 0.13. Next, 100 μL of bacterial suspension (~6.0 × 10^6^ to 1.0 × 10^7^) bacteria were administered to each mouse via tail vein injection. Mice were weighed at the time of challenge and every 24 h after, where infection was allowed to proceed for 4 days before mice were euthanized via cervical dislocation under anesthesia. Kidneys were aseptically harvested into 3 mL PBS with 0.1% (vol/vol) Triton X-100, homogenized, diluted, and plated onto TSA and MSA to enumerate bacterial burden.

To evaluate the virulence of the *perR*::Tn strain in a murine subcutaneous skin infection model, 8- to 10-week-old male and female BALB/c mice were shaved and depilated 1 day before infection. *S. aureus* strains were grown to the mid-exponential phase (OD_600_ of 2.0–2.5) in 25 mL TSB, washed twice with PBS, and resuspended in PBS to an OD_600_ of 1.4. Next, 50 μL of bacterial suspension roughly equal to 4.0 × 10^7^ CFUs was administered via subcutaneous injection to each flank. Infected mice were monitored daily for 2 days before being euthanized via cervical dislocation under anesthesia, 48 h post-infection. Tissues of approximately equal area/weight from each flank were harvested into 3 mL PBS with 0.1% (vol/vol) Triton X-100, homogenized, diluted, and plated onto MSA to enumerate bacterial burden. Lesion sizes were analyzed in ImageJ.

### Assessment of loss-of-function variation in *perR*

LOF variants were first assessed in *perR* across 8,375 clinical *S. aureus* isolates to evaluate disruptive variation in this regulator. To assess whether *perR* conservation was gene-specific, the same analysis was extended to additional transcriptional regulators (*fur*, *mntR*, *purR*, and *agrA*). LOF frequency was calculated as the proportion of isolates carrying at least one predicted LOF variant per gene, where LOF variants were defined as mutations resulting in premature stop codons or truncations shorter than 90% of the reference protein length. Binomial 95% confidence intervals were estimated using the Wilson score method. Statistical comparisons were performed using Fisher’s exact test with *perR* as a reference, and *P*-values were adjusted for multiple testing using the Benjamini–Hochberg procedure.

## Data Availability

Genomic sequence data for NJ4 have been deposited in the SRA database under BioProject PRJNA1423742. Raw RNA-seq reads have been deposited in the GEO database under accession GSE318095.

## SUPPLEMENTAL MATERIAL

Supplemental material jb.00149-26-s0001.docx Supplemental methods, Figures S1 to S5 and Tables S2 to S3.

Table S1 jb.00149-26-s0002.xlsx RNAseq raw data.

ASM does not own the copyrights to Supplemental Material that may be linked to, or accessed through, an article. The authors have granted ASM a non-exclusive, world-wide license to publish the Supplemental Material files. Please contact the corresponding author directly for reuse.
