# Supplementary material for: The oxidative stress regulator, PerR, is required for staphyloferrin B-mediated iron acquisition in Staphylococcus aureus
Source: J Bacteriol. 2026 Jul 14;208(8):e00149-26. doi: 10.1128/jb.00149-26 (PMC13492143; doi:10.1128/jb.00149-26)
Supplement: Supplementary file 1 [file jb.00149-26-s0001.docx]

**Sheikh *et al*., Supplementary Information**

**Figure S1. (A)** Results of Kirby-Bauer disc diffusion assay with 0.3% v/v hydrogen peroxide as measured by zone of inhibition (ZOI) radius on a lawn of wild-type (WT) or *perR* mutant (*perR*::Tn) *S. aureus* USA300-LAC harboring either empty vector (pEmpty) or pALC2073 containing the *perR* gene (p*perR*). **(B)** Growth of wild-type (WT) and *perR* mutant (*perR*::Tn) *S. aureus* USA300-LAC harboring either empty vector (pEmpty) or pALC2073 containing the *perR* gene (p*perR*) on tryptic soy agar (TSA) or TSA with 1µM bovine hemin containing 200ng/mL streptonigrin.

**Figure S2.** 24-hour growth of wild-type and *perR* mutant (*perR*::Tn) *S. aureus* USA300-LAC in Roswell Park Memorial Institute (RPMI) medium containing either no additives (SF), 25% v/v human serum (25%) or 50% v/v human serum (50%). Bars represent the mean OD_600nm_ reading ± standard deviation where each data point represents an individual biological replicate and each shape represents an individual serum donor. The data derive from three independent experiments and statistical significance was determined using Brown-Forsythe and Welch’s ANOVA with Dunnett’s T3 multiple comparisons test for A and B, and unpaired t-test with Welch’s correction for C. ns indicates no significance; *p<0.05, ****p<0.0001.

**Figure S3.** Volcano plot of differential gene expression between **(A)** *perR* mutant relative to wild-type *S. aureus* in Fe-replete growth media and **(B)** wild-type *S. aureus* grown in Fe-depleted relative to Fe-replete growth media. Horizontal dotted line represents p-value cutoff of 0.01; vertical dotted lines represent fold change cutoff of 2 in either direction. Red points represent genes with significantly increased expression, blue genes with significantly decreased expression, and grey genes below one or both cutoffs. **(C)** Fold change in *sbnA* transcript and **(D)** *sfaA* transcript of *perR* mutant relative to WT *S. aureus* USA300-LAC as measured through RT-qPCR and calculated via ΔΔC_T_ method. -Fe = iron-restricted conditions, +Fe = iron-replete conditions. Cells were grown to early exponential phase in TSB containing 2,2’-dipyridyl as described in methods and subject to addition of ferric chloride (iron-replete) or no intervention (iron-deplete). Bars represent the mean fold change in transcript as determined via ΔΔC_T_ method ± standard deviation where each data point represents an individual biological replicate. The data derive from two independent experiments and statistical significance was determined using an unpaired t-test with Welch’s correction. ns indicates no significance; *p<0.05

**Figure S4. (A)** Position of two putative PerR boxes near the *sbnA* promoter and their sequences, along with PerR box sequences from other *perR*-regulated genes. Alignment performed in Geneious using MAFFT algorithm. Sequence logo created from MAFFT alignment data using WebLogo (1). **(B)** EMSA gel with *ahpC* and *sbnA* promoter probes incubated with purified PerR protein. *sbnA* probe was incubated with PerR protein at concentrations of 1.6 µM, 320 nM, 64 nM, and 12.8 nm. Gel was imaged on LiCor to capture IR800 signal from probes and then stained with Coomassie Brilliant Blue and imaged on a white light transilluminator to visualize migrated PerR protein.

**Figure S5.** 24-hour growth of *S. aureus* USA300-LAC harboring mutations in *perR* (*perR*::Tn) and *fur* in iron-restricted Tris-minimal succinate (TMS) medium containing either no additives (-Fe) or 10 μM FeCl_3_ (+Fe). In A, the bars represent the mean OD600nm reading ± standard deviation where each data point represents an individual biological replicate. The data are derived from three independent experiments, and statistical significance was determined using Brown-Forsythe and Welch’s ANOVA with Dunnett’s T3 for multiple comparisons. ns, indicates no significance; **p<0.01, ****p<0.0001.

**Supplementary Methods**

**Deletion of cntKLM and sst gene deletions.** As mentioned in methods the pKOR1 plasmid was used to created gene deletions (2). To create the pKOR-Δ*sst* plasmid the primer pairs sstA-F-Attb1 with sstA-R-SacI and sstD-F-SacI with sstD-R-AttB2 (supplementary Table 2) were used to amplify regions of homology from the *S. aureus* genome that flank the locus targeted for deletion. The resulting amplicons were digested with SacI and ligated using T4 DNA ligase to create a linear fragment that was recombined into the parental pKOR1 plasmid using Gateway® BP Clonase™️ Enzyme Mix (Invitrogen). The desired construct was selected for in *E. coli* DH5⍺ in the presence of ampicillin and identified by PCR using the primers above and restriction digest analysis. To create the pKOR-Δ*cntKLM* plasmid, regions of homology that flank the region targeted for deletion were amplified using the primer pairs cntK-F-SalI with cntK-R-BglII and cntM-F-BglII with cntM-R-KpnI (supplementary Table 2). The resulting amplicons were digested with BglII and KpnI or SalI as appropriate. The parental pKOR1 plasmid was digested with SalI and KpnI and the vector backbone isolated by gel extraction. The digested amplicons and pKOR1 plasmid were ligated using T4 DNA ligase (New England Biolabs), transformed into *E. coli* DH5⍺, and the desired construct selected for in the presence of ampicillin. The desired pKOR-Δ*cntKLM* was identified using the cloning primers described above and restriction analysis.

To delete the *cntKLM* and *sstABCD* loci the pKOR-∆*cntKLM* and pKOR-Δ*sst* plasmids were passaged through *S. aureus* RN4220 and then introduced into target *S. aureus* USA300 derivatives by electroporation. The desired deletions after cultivation at 42 ℃ and screening for chloramphenicol sensitive colonies were first verified using PCR with primers annealing outside of deleted loci (supplementary Table 2) and then ultimately with whole genome sequencing. First, a Δ*cntKLM* mutant was created in WT using this method, and previously described *sfa* and *sbn* mutations (3) mobilized into this strain to generate the *sbn sfa cnt* mutant strain. In this background, the pKOR-Δ*sst* plasmid was used to delete the *sst* locus.

**Detection of HtsA and SirA by western blot.** In brief, *S. aureus* was cultured overnight in TMG with and without the addition of 10 µM FeCl_3_, then cells were pelleted and lysed. Following lysis, samples were mixed with Laemmli buffer and boiled at 100 °C for 5 min. 10 µL of each sample, were loaded into a 1.5 mm SDS-PAGE gel with 4% stacking and 12% resolving sections and run at 150 V for 1.5 h. Electrophoresed proteins were transferred to a nitrocellulose membrane using a TransBlot Turbo (Bio-Rad) at 25 V and 1.0 A for 30 min. Membranes were blocked for 2 h with 10% (w/v) skim milk in PBS with 0.1% (v/v) Tween 20 (PBS-T), then incubated with rabbit anti-SirA or anti-HtsA primary antibody (1:1000) in blocking buffer overnight at 4 °C. The following day, the membrane was washed with PBS-T and incubated for 1 hr with goat anti-rabbit secondary antibody (Li-Cor) diluted to 1:20000 in blocking buffer then washed. Blots were imaged on an Odyssey CLx (Li-Cor) with emission filter set to 800 nm.

**Isolation of human serum.** Whole human blood was drawn from healthy adult volunteers upon verbal consent in accordance with protocols approved by Western University’s Research Ethics Board, London, Ontario Canada. Blood was collected into non-heparinized serum vacutainer tubes (BD), allowed to rest at room temperature for 30 min to coagulate, and then serum separated by centrifugation at 4°C for 10 min at 1000 × *g*.

**Purification of recombinant PerR protein.** To obtain recombinant PerR protein, the *perR* gene from *S. aureus* USA300 was cloned into pET28a to be in-frame with the N-terminal hexahistidine tag. BL21 *E. coli* harboring the pET28a-PerR plasmid were grown to an OD_600_ of 0.6 in 2L of LB broth containing 30 µg/mL kanamycin and 44µM 2,2’-dipyridyl, induced with 0.5mM IPTG, and left to grow overnight at 37℃ with shaking. Cells were pelleted and resuspended in binding buffer (20 mM Tris-HCl, 150 mM NaCl, 2 mM imidazole), mechanically lysed, and the lysate passed through a HisPur cobalt resin column (Thermo Scientific). Following a wash with buffer containing 20 mM Tris-HCl, 150 mM NaCl, and 20 mM imidazole, hexahistidine-tagged PerR was eluted from the column using elution buffer (20 mM Tris-HCl, 150 mM NaCl, and 200 mM imidazole). All buffers were treated with Chelex-100 resin (Bio-RAD) according to manufacturer’s instructions to remove trace divalent metals that might bind to PerR and catalyze its oxidation.

**Electrophoretic mobility shift assays (EMSAs).** For EMSAs, IR800-tagged PCR primers were designed to amplify the promoter regions of the USA300 *ahpC* and *sbnA* genes, ensuring the inclusion of putative PerR boxes **(supplementary Table 2)**. Probes were amplified using conventional PCR with these primers and *S. aureus* USA300 gDNA as template, then purified using an enzymatic cleanup kit (Omega) according to manufacturer’s instructions.

EMSAs were performed in binding reactions consisting of 5uL binding buffer (30mM Tris-HCl pH 8.0, 120 mM KCl, 1 mM MgCl_2_, 1 mM MnCl_2_, 20% glycerol v/v), 1 uL poly(dI-dC) (100 ng/uL), purified recombinant thrombin-cleaved PerR at concentrations ranging from 12.8 nM to 1.6 µM, 100 ng of IR800-tagged nucleic acid probe, and water up to 25 uL. All components except the probe were added to reaction tubes, left to incubate at room temperature for 30 min, then probe was added and mixture incubated for an additional 1 h. Next, samples were run on non-denaturing TBE-acrylamide gel (45 mM Tris-borate, 6% acrylamide, 2.5% glycerol) with TBE running buffer at 120V for 45 min. Gels were imaged first on an Odyssey CLx (Li-Cor) with emission filter set to 800 nm, then stained with Coomassie Brilliant Blue and imaged on a GelDoc Go (Biorad).

**Table S2. Bacterial strains and plasmids used in this study.**

| **Bacterial strains or plasmids** | **Description** | **Source or reference** |
| --- | --- | --- |
| **Strains** |  |  |
| *E. coli* |  |  |
| DH5α | F^-^ (λ^-^) (φ80ΔlacZΔM15) Δ(lacZYA-argF) U169 glnV44 deoR gyrA96 recA1 relA91 endA1 thi-1 hsdR17 | Laboratory stock |
| BL21 | F^-^ ompT gal dcm lon hsdS_B_ (r_B_^–^ m_B_^–^) λ(DE3 [lacI lacUV5-T7 gene 1 ind1 sam7 nin5]) | Laboratory stock |
| *S. aureus* |  |  |
| USA300 | USA300 LAC; hypervirulent community-associated MRSA; cured of antibiotic resistance plasmid | Laboratory stock |
| RN4220 | r_K_^-^ m_K_^-^ ; capable of accepting foreign DNA | (4) |
| Newman | Wild-type clinical osteomyelitis isolates | (5) |
| Δ5 | Derivative of *S. aureus* USA300 with *sbn*::Tc, *sfa*::Km, *fhuG*::Ery, Δ*cntKLM*, and Δ*sst*; Ery^r^ Km^r^ Tc^r^ | This study |
| NJ4 | Streptonigrin-heme resistant derivative of Δ5 with Y66X SNP in *perR*; Ery^r^ Km^r^ Tc^r^ | This study |
| *perR::Tn* mutant | Derivative of *S. aureus* USA300 with *perR*::φNΣ transduced from the Nebraska transposon library; Ery^r^ | This study |
| *sbn/sfa/cnt* mutant | Derivative of *S. aureus* USA300 with *sbn*::Tc, *sfa*::Km, and ΔcntKLM; Km^r^ Tc^r^ | This study |
| *sbn* mutant | Derivative of *S. aureus* USA300 with *sbn*::Tc; Tc^r^ | (3) |
| *sfa* mutant | Derivative of *S. aureus* USA300 with *sfa*::Km; Km^r^ | (3) |
| *sbn sfa* mutant | Derivative of *S. aureus* *sbn* mutant with *sfa*::Km; Km^r^ Tc^r^ | (3) |
| *sbn perR* mutant | Derivative of *S. aureus* *sbn* mutant with *perR*::φNΣ transduced from the Nebraska transposon library; Ery_R_ Tc_R_ | This study |
| *sfa perR* mutant | Derivative of *S. aureus* *sfa* mutant with *perR*::φNΣ transduced from the Nebraska transposon library; Ery^r^ Km^r^ | This study |
| *sbn sfa perR::Tn* mutant | Derivative of *S. aureus* *sbn sfa* mutant with *perR*::φNΣ transduced from the Nebraska transposon library; Ery^r^ Km^r^ Tc^r^ | This study |
| *sirA* mutant | Derivative of *S. aureus* Newman with *sirA*::Km; Km^r^ | (6) |
| *hts* mutant | Derivative of *S. aureus* Newman with *hts*::Tc; Tc^r^ | (7) |
| *sbn sir* mutant | Derivative of *S. aureus* Newman with *sbnA*::Km and sirB::Tc; Km^r^ Tc^r^ | (7, 8) |
| *sbn hts* mutant | Derivative of *S. aureus* Newman with *sbnE*::Km and hts::Tc; Km^r^ Tc^r^ | (7, 8) |
| *fur* mutant | Derivative of *S. aureus* USA300 with *fur*::Km; Km^r^ | (7) |
| *perR fur* mutant | Derivative of *fur* mutant with *perR*::φNΣ transduced from the Nebraska transposon library; Ery^r^ Km^r^ | This study |
| *perR fur sbn* mutant | Derivative of *perR fur* mutant with *sbn*::Tc transduced from *sbn* mutant; Ery^r^ Km^r^ Tc^r^ | This study |
| *agr* mutant | Derivative of *S. aureus* USA300 with agr::Tc; Tc^r^ | (9) |
| **Plasmids** |  |  |
| pALC2073 | *E. coli*/*S. aureus* shuttle vector; Ap^r^ Cm^r^ | (10) |
| pPerR | pALC2073 with perR from *S. aureus* USA300; Ap^r^ Cm^r^ | This study |
| pKOR1 | *S. aureus* allelic exchange vector; Ap^r^ Cm^r^ | (2) |
| pKOR-Δ*cntKLM* | pKOR1 with upstream and downstream regions of homology to *cntKLM* | This study |
| pKOR-Δ*sst* | pKOR1 with upstream and downstream regions of homology to *sstABCD* | This study |
| pET28α | Overexpression vector for 6x histidine-tagged proteins; Km^r^ | Novagen |
| pET28α-perR | pET28α derivative encoding N-terminally hexahistidine-tagged PerR from *S. aureus* USA300; Km^r^ | This study |

**Table S3. Oligonucleotides used in this study.**

| Purpose | Name | Sequence (5'-3') |
| --- | --- | --- |
| Cloning primers for perR into pALC | KpnI-perR-F | TATATTGGTACCATTATATAAGAAAGATGGTGATATAG |
|  | EcoRI-perR-R | TATATAGAATTCCAAAGTTAAATTATTTATCTTGGC |
| Cloning primers for perR into pET28 | NheI-perR-F | CAGTCACGCTAGCATGAGTGTTGAAATAGAATCAATTG |
|  | BamHI-perR-R | CTGACTGGATCCTTATTTATCTTGGCATTCTTTACAAAC |
| Cloning primers for fur into pET28 | NheI-fur-F | CTGACTGCTAGCTTGGAAGAACGATTAAATCGC |
|  | BamHI-fur-R | CTGACAGGATCCCTATCCTTTACCTTTAGCTTGG |
| PCR confirmation of fur deletion | fur-F | TGTAATGATTTTAATTATTAGTAGG |
|  | fur-R | GAAGTTAATTAACCTACGCA |
| PCR confirmation of perR transposon mutation | perR-seqR | GGTGATATAGATGAGTGTTGAA |
|  | upstream | CTCGATTCTATTAACAAGGG |
| PCR confirmation of sbn deletion | sbn-F | AACAACACGCTTTGGTTTCC |
|  | sbn-R | AGAAACTCTGCCCAAAGCAA |
| qPCR for sbnA gene | qPCR-sbn-F | CATGGTGCAGGCACAGAGATTG |
|  | qPCR-sbn-R | ATACCCATAATGCTACCTGTCGTGC |
| qPCR for sfaA gene | qPCR-sfa-F | GCACTTAAAATCCCTCTTAATGTCGC |
|  | qPCR-sfa-R | TACTAAGTGTGTGGGCAGGG |
| Generation of IR800-tagged ahpC EMSA probe | ahpC-probe-F | AATGACATAAATATCTTCCTCCTAAG |
|  | ahpC-probe-R | /IRD800/CTACAAAATCCATTATCAAAGCAC |
| Generation of IR800-tagged sbnA EMSA probe | sbnA-probe-F | GAGAATCATTATCACGATTTAGT |
|  | sbnA-probe-R | /IRD800/AATGAATCGTGACATGC |
| Cloning primers for pKOR-Δsst | sstA-F-Attb1 | GGGGACAAGTTTGTACAAAAAAGCAGGCTTATTTATTAAAGGGAAATATTTTGC |
|  | sstA-R-SacI | TTTTTTGAGCTCCTAACATCATCGTATGAGGGATAGCA |
|  | sstD-F-SacI | TTTTTTGAGCTCGCTGCGACGAAACCCGAAGTAATC |
|  | sstD-R-AttB2 | GGGGACCACTTTGTACAAGAAAGCTGGGTCAACTTTATCAAGTTCCTCAATTTG |
| Cloning primers for pKOR-ΔcntKLM | cntK-F-SalI | TTTTTTGTCGACATGAATCGGCAGGTTATAGAAT |
|  | cntK-F-SalI | TTTTTTGTCGACATGAATCGGCAGGTTATAGAAT |
|  | cntK-R-BglII | TTTTTTAGATCTCTGGTATATAGATTAATGGCTG |
|  | cntM-F-BglII | TTTTTTAGATCTGTTGATAAAGAAGCGCCTAATCATGTG |
|  | cntM-R-KpnI | TTTTTTGGTACCTTATGAAAGCGTTCTATTGATTTC |
| PCR confirmation of sst deletion | Δsst-F | CAAGATGATGACTTTGTATTTGACAAC |
|  | Δsst-R | AGGAATCGTAAGTTAAGGAGCTTTGTC |
| PCR confirmation of cntKLM deletion (with cntM-R-KpnI) | cnt-check | CCTCTAGTAATTAAAATTACAGAGG |
| Purpose | Name | Sequence (5'-3') |
| Cloning primers for perR into pALC | KpnI-perR-F | TATATTGGTACCATTATATAAGAAAGATGGTGATATAG |
|  | EcoRI-perR-R | TATATAGAATTCCAAAGTTAAATTATTTATCTTGGC |
| Cloning primers for perR into pET28 | NheI-perR-F | CAGTCACGCTAGCATGAGTGTTGAAATAGAATCAATTG |
|  | BamHI-perR-R | CTGACTGGATCCTTATTTATCTTGGCATTCTTTACAAAC |
| Cloning primers for fur into pET28 | NheI-fur-F | CTGACTGCTAGCTTGGAAGAACGATTAAATCGC |
|  | BamHI-fur-R | CTGACAGGATCCCTATCCTTTACCTTTAGCTTGG |
| PCR confirmation of fur deletion | fur-F | TGTAATGATTTTAATTATTAGTAGG |
|  | fur-R | GAAGTTAATTAACCTACGCA |
| PCR confirmation of perR transposon mutation | perR-seqR | GGTGATATAGATGAGTGTTGAA |
|  | upstream | CTCGATTCTATTAACAAGGG |
| PCR confirmation of sbn deletion | sbn-F | AACAACACGCTTTGGTTTCC |
|  | sbn-R | AGAAACTCTGCCCAAAGCAA |
| qPCR for sbnA gene | qPCR-sbn-F | CATGGTGCAGGCACAGAGATTG |
|  | qPCR-sbn-R | ATACCCATAATGCTACCTGTCGTGC |
| qPCR for sfaA gene | qPCR-sfa-F | GCACTTAAAATCCCTCTTAATGTCGC |
|  | qPCR-sfa-R | TACTAAGTGTGTGGGCAGGG |
| Generation of IR800-tagged ahpC EMSA probe | ahpC-probe-F | AATGACATAAATATCTTCCTCCTAAG |
|  | ahpC-probe-R | /IRD800/CTACAAAATCCATTATCAAAGCAC |
| Generation of IR800-tagged sbnA EMSA probe | sbnA-probe-F | GAGAATCATTATCACGATTTAGT |
|  | sbnA-probe-R | /IRD800/AATGAATCGTGACATGC |
| Cloning primers for pKOR-Δsst | sstA-F-Attb1 | GGGGACAAGTTTGTACAAAAAAGCAGGCTTATTTATTAAAGGGAAATATTTTGC |
|  | sstA-R-SacI | TTTTTTGAGCTCCTAACATCATCGTATGAGGGATAGCA |
|  | sstD-F-SacI | TTTTTTGAGCTCGCTGCGACGAAACCCGAAGTAATC |
|  | sstD-R-AttB2 | GGGGACCACTTTGTACAAGAAAGCTGGGTCAACTTTATCAAGTTCCTCAATTTG |
| Cloning primers for pKOR-ΔcntKLM | cntK-F-SalI | TTTTTTGTCGACATGAATCGGCAGGTTATAGAAT |
|  | cntK-F-SalI | TTTTTTGTCGACATGAATCGGCAGGTTATAGAAT |
|  | cntK-R-BglII | TTTTTTAGATCTCTGGTATATAGATTAATGGCTG |
|  | cntM-F-BglII | TTTTTTAGATCTGTTGATAAAGAAGCGCCTAATCATGTG |
|  | cntM-R-KpnI | TTTTTTGGTACCTTATGAAAGCGTTCTATTGATTTC |
| PCR confirmation of sst deletion | Δsst-F | CAAGATGATGACTTTGTATTTGACAAC |
|  | Δsst-R | AGGAATCGTAAGTTAAGGAGCTTTGTC |
| PCR confirmation of cntKLM deletion (with cntM-R-KpnI) | cnt-check | CCTCTAGTAATTAAAATTACAGAGG |

**Supplementary References**

1.	Crooks GE, Hon G, Chandonia J-M, Brenner SE. 2004. WebLogo: A Sequence Logo Generator. Genome Res 14:1188–1190.

2.	Bae T, Schneewind O. 2006. Allelic replacement in *Staphylococcus aureus* with inducible counter-selection. 1. Plasmid 55:58–63.

3.	Hannauer M, Sheldon JR, Heinrichs DE. 2015. Involvement of major facilitator superfamily proteins SfaA and SbnD in staphyloferrin secretion in Staphylococcus aureus. 6. FEBS letters 589:730–7.

4.	Kreiswirth BN, Löfdahl S, Betley MJ, O’Reilly M, Schlievert PM, Bergdoll MS, Novick RP. 1983. The toxic shock syndrome exotoxin structural gene is not detectably transmitted by a prophage. 5936. Nature 305:709–712.

5.	Duthie ES, Lorenz LL. 1952. Staphylococcal coagulase; mode of action and antigenicity. 1–2. Journal of general microbiology 6:95–107.

6.	Dale SE, Sebulsky MT, Heinrichs DE. 2004. Involvement of SirABC in iron-siderophore import in Staphylococcus aureus. 24. J Bacteriol 186:8356–62.

7.	Beasley FC, Vinés ED, Grigg JC, Zheng Q, Liu S, Lajoie GA, Murphy MEP, Heinrichs DE. 2009. Characterization of staphyloferrin A biosynthetic and transport mutants in *Staphylococcus aureus*. Mol Microbiol 72:947–963.

8.	Beasley FC, Marolda CL, Cheung J, Buac S, Heinrichs DE. 2011. *Staphylococcus aureus* transporters Hts, Sir, and Sst capture iron liberated from human transferrin by Staphyloferrin A, Staphyloferrin B, and catecholamine stress hormones, respectively, and contribute to virulence. 6. Infect Immun 79:2345–55.

9.	Flannagan RS, Kuiack RC, McGavin MJ, Heinrichs DE. 2018. *Staphylococcus aureus* uses the GraXRS regulatory system to sense and adapt to the acidified phagolysosome in macrophages. 4. mBio 9:e01143-18.

10.	Bateman BT, Donegan NP, Jarry TM, Palma M, Cheung AL. 2001. Evaluation of a tetracycline-inducible promoter in *Staphylococcus aureus* in vitro and in vivo and its application in demonstrating the role of *sigB* in microcolony formation. 12. Infection and immunity 69:7851–7857.
